# Enhanced object detection in remote sensing images by applying metaheuristic and hybrid metaheuristic optimizers to YOLOv7 and YOLOv8

**DOI:** 10.1038/s41598-025-89124-8

**Published:** 2025-02-28

**Authors:** Khaled Mohammed Elgamily, M. A. Mohamed, Ahmed Mohamed Abou-Taleb, Mohamed Maher Ata

**Affiliations:** 1 Department of Electronics and Communications Engineering, Faculty of Engineering, Mansoura University, Mansoura, 35516 Egypt; 2 School of Computational Sciences and Artificial Intelligence (CSAI), Zewail City of Science and Technology, October Gardens, 6th of October City, Giza, 12578 Egypt

**Keywords:** Object detection, YOLOv7, YOLOv8, Hybrid metaheuristic optimization, Optimization techniques, Remote sensing images, Computer science, Information technology, Engineering, Aerospace engineering, Electrical and electronic engineering

## Abstract

Developments in object detection algorithms are critical for urban planning, environmental monitoring, surveillance, and many other applications. The primary objective of the article was to improve detection precision and model efficiency. The paper compared the performance of six different metaheuristic optimization algorithms including Gray Wolf Optimizer (GWO), Particle Swarm Optimization (PSO), Genetic Algorithm (GA), Remora Optimization Algorithm (ROA), Aquila Optimizer (AO), and Hybrid PSO–GWO (HPSGWO) combined with YOLOv7 and YOLOv8. The study included two distinct remote sensing datasets, RSOD and VHR-10. Many performance measures as precision, recall, and mean average precision (mAP) were used during the training, validation, and testing processes, as well as the fit score. The results show significant improvements in both YOLO variants following optimization using these strategies. The GWO-optimized YOLOv7 with 0.96 mAP 50, and 0.69 mAP 50:95, and the HPSGWO-optimized YOLOv8 with 0.97 mAP 50, and 0.72 mAP 50:95 had the best performance in the RSOD dataset. Similarly, the GWO-optimized versions of YOLOv7 and YOLOv8 had the best performance on the VHR-10 dataset with 0.87 mAP 50, and 0.58 mAP 50:95 for YOLOv7 and with 0.99 mAP 50, and 0.69 mAP 50:95 for YOLOv8, indicating greater performance. The findings supported the usefulness of metaheuristic optimization in increasing the precision and recall rates of YOLO algorithms and demonstrated major significance in improving object recognition tasks in remote sensing imaging, opening up a viable route for applications in a variety of disciplines.

## Introduction

A crucial part of many applications, including urban planning, environmental monitoring, and surveillance, is object detection in remote sensing imagery^1^. Convolutional neural networks (CNNs) have demonstrated impressive results in object detection tasks. The YOLO (You Only Look Once) architecture is particularly notable for its accuracy and efficiency. The most recent object detection models, YOLOv7 and YOLOv8, have outperformed their predecessors in tests. With the introduction of trainable bag-of-freebies and re-parameterization approaches, YOLOv7 enhances performance by lowering the number of parameters and computation^2^. On the other hand, YOLOv8, one of the most recent versions of the YOLO series outperforms earlier YOLO models in terms of accuracy and speed^3^. These advancements in the YOLO family have contributed to improving object detection capabilities in various domains, including remote sensing images. The implementation of YOLO in remote sensing provides the advantages of accurate and effective detection of objects, making it a critical instrument for urban planning, environmental monitoring, and surveillance. Nevertheless, optimizing performance necessitates a complicated and intricate process of fine-tuning several hyperparameters. Metaheuristic optimization techniques present a viable method for effectively navigating the extensive hyperparameter space and identifying the best neural network configurations^4^. These methods have been widely used in a variety of optimization issues because of their efficacious handling of highly dimensional, non-linear, and irregular search spaces, which draw inspiration from natural or social phenomena. Furthermore, a multitude of optimization problems have shown that hybrid metaheuristic approaches—which blend several optimization strategies—perform better in some object detection tasks^5^. The problem this study attempts to solve is the challenging task of significantly improving detection precision and model efficiency in object detection by utilizing the capabilities of metaheuristic and hybrid metaheuristic optimization strategies with the YOLOv7 and YOLOv8 algorithms, particularly in the context of remote sensing images. To successfully negotiate the complexity of the optimization process and make notable improvements in object identification performance, this undertaking requires careful exploration and committed efforts. The main steps of the proposed system are (i) data preprocessing and augmentation, (ii) YOLO model training and optimization, and (iii) object detection. The major contributions of this article are:

1. Proposing enhanced object detection in remote sensing images by applying metaheuristic and hybrid metaheuristic optimizers to YOLOv7 and YOLOv8.
2. Utilizing the performance impact of different metaheuristic optimization models on the YOLO algorithm detection process of objects in remote sensing images.
3. Comparing the performance of the combination of different metaheuristic optimizers and YOLO structures.

The rest of this article is organized as follows: The literature review is summarized in section "Related work". Section "Materials and methods" provides a detailed analysis of the applied approaches and algorithms. The experimental results of the proposed method are presented in section "Experimental results". Finally, the research is concluded in section "Conclusion".

## Related work

### Object detection

Li et al.^6^ described developments in object detection using benchmark datasets and cutting-edge deep learning techniques in artificial intelligence and satellite observation. They presented a comprehensive, open-access object detection benchmark dataset designed to support the earth observation community’s ongoing investigation and verification of deep learning techniques. They utilized this dataset to evaluate representative object detection techniques to create a useful performance baseline.

Han et al.^7^ presented the Single-Shot Alignment Network (S2A-Net) to detect aligned objects in remote sensing photos. The study accomplished a complete feature alignment and resolved discrepancies between classification and regression, it integrated an orientation detection mechanism and feature alignment algorithm. The method also investigated detection on large-scale images to maximize the trade-off between speed and accuracy. Comprehensive tests on the DOTA and HRSC2016 datasets demonstrated that S2A-Net performs at the cutting edge, proving its efficacy in the detection of flying objects.

Cheng et al.^8^ presented FENet, a novel technique for multiple classes detection in satellite images to address complex backdrop circumstances and sparse item distributions. A Dual Attention Feature Enhancing algorithm built into FENet helped to steer robust object detection by selecting and emphasizing useful attributes from various resolutions. Furthermore, enhanced context Features technique bridged multiscale feature maps by utilizing global as well as local contextual data. experimental results on DIOR and DOTA datasets illustrated the FENet algorithm’s effectiveness for object detection purposes.

Feng et al.^9^ presented an optimized lightweight Yolov5s-based target detection algorithm for winter jujube detection. Yolov5’s backbone net is replaced with Shufflenet V2, which significantly reduces dimensions and parameters. Furthermore, Yolov5’s original neck structure is replaced with a slim neck using GSConv and VoV-GSCSP to improve accuracy while simplifying the model.

Tian et al.^10^ proposed KCFS-YOLOv5, an object detection technique for satellite images. The article applied K-means to optimize anchor selection, merge Coordinate Attention (CA) with the backbone of YOLOv5’s architecture, introduce a Bidirectional Feature Pyramid Network (BiFPN) into the neck network, and create a micro-object detection unit to solve issues in satellite imagery. Furthermore, to minimize the difference between the ground truth and forecast boxes, they utilized the SIoU Loss function. The accuracy of KCFS-YOLOv5 in detection is proven by examination of the NWPU VHR-10, RSOD, and UCAS-AOD-CAR datasets.

Ouyang-Zhang et al.^11^ utilized the detection Transformer (DETR) framework to enable end-to-end object identification by immediately transforming demands into distinct objects. They carried out an investigation comparing the one-to-many label mappings in classic detectors employing non-maximum suppression (NMS) with the one-to-one matching in DETRs. Their findings consistently showed that great performance in detecting transformers can be achieved without bipartite matching.

Fu etal.^12^ improved small object detection with YOLOv7-Drone, designed for remote sensing imagery, by swapping out the P5 detection head for the P2 detection head. Efficient information flow was ensured by optimizing feature transmission through the CBS module’s network optimization. To minimize unnecessary calculations, the TGM-CESC module limited the sparse masks of the convolution at the level of pixels and directed the architecture only toward foreground objects. To prevent the loss of data, the HCEM technique was included.

Shen et al.^13^ utilized an adaptive CNN to refine the lightweight DCN_C2f. module for P3 and P4 identification, they improved YOLOv8 for real-time object detection in remote sensing photos. their improvement enhances the capture of item size and shape, especially for obstructed and multi-scale objects. Furthermore, to mitigate information loss and enable featured self-calibration across various scales, they presented the Self-Calibrating Shuffle Attention (SC_SA) mechanism. The experimental results show the performance of DS-YOLOv8, which achieves great accuracy.

Wang et al.^14^ presented the RSSOD dataset for remote sensing object detection focusing on small objects and challenging situations. the study suggested using the RFA-based MCGR network to accomplish object detection tasks and obtain cutting-edge picture high-resolution (SR) quality.

Qin et al.^15^ Introduced an enhanced Faster R-CNN, by utilizing DarkNet53, which uses skip connections to tackle the disappearance of gradients in deep networks, in place of the two primary feature extraction networks of the Faster R-CNN technique, ResNet50, and VGG16. Furthermore, they utilized data augmentation to tackle the problem of inadequate landslip samples within the applied remote sensing training set.

Qian et al.^16^ presented an enhanced boundary box regressor and multi-level combined features technique for ODRSIs. The article presented IGIoU loss for adaptive optimization, MLFF for improved feature extraction, and GIoU for parallel scenarios. Significant enhancements in performance are shown by the evaluation of the DIOR and NWPU VHR-10 datasets. When compared to other approaches, the method delivers state-of-the-art performance and great accuracy in object localization.

Diwan et al.^17^ represented an extensive overview of single-stage object detectors, with a focus on YOLOs in particular, emphasizing structural innovations, evaluations of performance, and regression construction. The article contrasted the inference time and detection accuracy, demonstrating YOLO’s quicker inference speed despite its marginally poorer accuracy when compared to two-stage detectors such as Fast-RCNN. The review highlights the importance of YOLO’s speed advantages in a variety of applications.

Yi et al.^18^ Introduced LAR-YOLOv8, an enhanced satellite imaging algorithm that adds transformation blocks and a two-branch attention system to the local module. Discriminative characteristics are improved by a bidirectional features pyramidal structure that is attention guided. The article compared LAR-YOLOv8 with YOLOv8 on the NWPU VHR-10, RSOD, and CARPK datasets. When compared to traditional and remote sensing detection networks, the utilized approach showed good results in terms of mAP (small), mAP, parameters, and FPS, significantly improving small target recognition in satellite photos.

Adegun et al.^19^ analyzed aerial information obtained from satellites across a variety of landscapes using multi-object identification deep learning methods in conjunction with transfer learning. Five techniques based on YOLO and R-CNN were evaluated. Detailed evaluations of performance are included in the results, and for vegetation and swimming pools, detection accuracies above 90%. At 0.2 ms, YOLOv8 demonstrated the fastest detection speed. A further validation of the model’s efficacy is conducted using the Visdron and PASCAL VOC2007 datasets.

Gomroki et al.^20^ propose a 3-D building change detection approach that employs an encoder-decoder network based on YOLOv7 to accurately map numerous building modifications. The procedure entails preprocessing with mixed augmentation, network training, and prediction. The technique, which has been tested on UAV and satellite datasets from Mashhad and Tehran, can manage highly imbalanced data while improving detection accuracy. The findings make a substantial contribution to the object detection literature, showing YOLOv7’s potential in remote sensing applications. Table 1 summarizes the most recent research articles that discuss the detection of multiple objects in remote-sensing images.

**Table 1:** Complete summary of the literature review of recent research articles in state of art of remote sensing image object detection.

Authors	Algorithm/model	Strengths	Challenges
Li et al.^6^	Object Detection Benchmark	Comprehensive, open-access dataset for earth observation community	Requires continuous updates to remain relevant with evolving techniques
Han et al.^7^	S2A-Net	Complete feature alignment, orientation detection, and high performance on DOTA and HRSC2016 datasets	Balancing speed and accuracy on large-scale images
Cheng et al.^8^	FENet	Dual Attention Feature Enhancing, effective in complex backdrops	Handling sparse item distributions in satellite images
Feng et al.^9^	Optimized Yolov5s	Lightweight, improved accuracy with Shufflenet V2 and GSConv	Maintaining model simplicity while enhancing accuracy
Tian et al.^10^	KCFS-YOLOv5	K-means optimized anchors, BiFPN, SIoU Loss function, and high accuracy	Addressing micro-object detection challenges in satellite imagery
Ouyang-Zhang et al.^11^	DETR Framework	End-to-end object identification, no need for bipartite matching	Comparing performance with traditional detectors using NMS
Fu et al.^12^	YOLOv7-Drone	Improved small object detection, and efficient information flow	Minimizing unnecessary calculations while focusing on foreground objects
Shen et al.^13^	DS-YOLOv8	Adaptive CNN, Self-Calibrating Shuffle Attention, high accuracy	Enhancing real-time detection for obstructed and multi-scale objects
Wang et al.^14^	RFA-based MCGR Network	High-resolution image quality, effective for small-sized objects	Handling challenging situations in remote sensing object detection
Qin et al.^15^	Enhanced Faster R-CNN	Uses DarkNet53, data augmentation for landslip samples	Tackling gradient disappearance in deep networks
Qian et al.^16^	Enhanced Boundary Box Regressor	Applying IGIoU loss, MLFF, GIoU,	Improving object localization accuracy
Diwan et al.^17^	Single-Stage Object Detectors	Emphasis on YOLO’s speed advantages, and structural innovations	Balancing inference speed with detection accuracy
Yi et al.^18^	LAR-YOLOv8	Transformation blocks, two-branch attention system, high mAP and FPS	Enhancing small target recognition in satellite photos
Adegun et al.^19^	Multi-Object Identification	High detection accuracy for vegetation and swimming pools, fast detection speed	Validating model efficacy across diverse landscapes
Gomroki et al.^20^	Encoder-decoder network based on YOLOv7	Accurately maps numerous building modifications; handles highly imbalanced data	Managing highly unbalanced datasets; achieving accurate multiple change detection at an acceptable speed

### Optimization techniques

Rajwar et al.^21^ addressed the topic of significant similarities amongst over 540 metaheuristic architectures (MAs) and provided a thorough assessment of them. It also tracks the proliferation of MAs. It casts doubt on the uniqueness of optimization strategies using altered search parameters. A new taxonomy that classifies MAs according to control parameters is presented. Future research directions are illuminated by the discussion of open challenges and real-world applications. The study intends to be a useful tool for the research community by helping novices comprehend metaheuristics and their applications.

Abd Elaziz et al.^22^ presented a comprehensive assessment of modern optimization techniques, including swarm intelligence (SI) and evolutionary computing (EC), used to improve the accuracy of deep neural network (DNN) achievement on a range of applications. The study highlighted how important optimization methods are for figuring out the best hyperparameters and DNN structures when working with big datasets. The work demonstrated how well meta-heuristic techniques optimize DNN models and how they may enhance effectiveness across a variety of scenarios.

Dey et al.^23^ proposed a meta-heuristic feature selection (FS) approach called The Golden Ratio-based Equilibrium Optimization (GREO) algorithm, which combines Golden Ratio Optimization (GRO) and Equilibrium Optimization (EO). These chosen characteristics are inputted into an XGBoost classification algorithm, which uses speech analysis to classify emotions. GREO surpasses component algorithms and prior FS approaches, achieving outstanding accuracy on SAVEE and EmoDB datasets, respectively. The model demonstrated the effectiveness of meta-heuristic techniques in selecting features for speech analysis tasks by providing a less costly computational solution for emotion categorization.

Myriam et al.^24^ suggested a reliable method for identifying cancer of the oral cavity in medical photographs by combining an optimized deep network (DBN) with a network of convolutional neural networks (CNN). A hybrid Particle Swarm Optimization (PSO) and Al-Biruni Earth Radius (BER) Optimization algorithm (PSOBER) are used to optimize the design parameters. Using a common biomedical dataset, the suggested strategy surpasses current methods with great accuracy. The approach’s stability and importance are confirmed by statistical tests.

Zhang et al.^25^ presented a hybrid optimization method, called AOAAO to overcome the shortcomings of current algorithms, which combines the Arithmetic Optimization algorithm (AOA) and the Aquila Optimizer (AO). A piecewise linear map is added to reduce parameter unpredictability, and the energy factor E is provided to regulate the use and exploration of AOAAO swarms, based on the success of the Harris Hawk optimization (HHO) method. Simulation investigations on engineering issues and reference variables showed that AOAAO is more efficient than nine well-known algorithms in optimized performance, with more rapid convergence rates and greater precision.

Sohlot et al.^26^ ensured the lowest environmental impact by optimizing crop fertilizer spraying through the use of hybrid OBHS and MRFO algorithms. While MRFO, which is motivated by the foraging behaviors of manta rays, effectively tackles real-world engineering challenges, OBHS speeds up convergence. Exhibiting outstanding efficiency with a great precision of 99% in fertilizer optimization, the outcomes signify a noteworthy breakthrough in this domain. Through the integration of these two methodologies, the research presented a new methodology.

Baghdadi et al.^27^ applied neural networks to diagnostic imaging to address the crucial need for reliable identification of breast cancer. The article suggested using MRFO optimization and transfer learning to improve a CNN-based system for classifying ultrasound and histology data. The system demonstrates its potential for accurate breast cancer diagnosis by outperforming existing approaches, as seen by its outstanding accuracy rates of 99.01%.

Wang et al.^28^ combined Aquila Optimizer (AO) and Harris Hawks Optimizer (HHO) to create a novel optimization algorithm called IHAOHHO. To improve its exploration and extraction capabilities, the article combined a random opposition-based learning technique with a nonlinear escape energy factor. The extensive investigations on technological issues and benchmark measures showed that IHAOHHO performed better than traditional AO, HHO, as well as other cutting-edge algorithms.

Nguyen et al.^29^ discussed the importance of monitoring soil salinity in the Ben Tre province of Vietnam to guarantee food security. Precision is greatly improved by combining optimization algorithms (HHO, SSA, MSA, BSA, GWO, and PSO) with the XGR model, outperforming traditional models.

Wen et al.^30^ proposed an enhanced Remora Optimization Algorithm (ROA) by utilizing Lens Opposition-Based Learning (LOBL) with ROA called IROA. Inspired by concepts from optical lens imaging, LOBL improved ROA’s efficiency by helping it escape local optima. IROA outperforms five existing meta-heuristic approaches in tackling optimization challenges, as demonstrated by experimental validation over 13 benchmark functions and automotive crashworthiness design.

Agushaka et al.^31^ discussed the crucial role that initialization plays in population-based metaheuristic methods. A variety of initialization strategies are reviewed within the article. The study assessed their efficacy and provided ideas for more investigation. Furthermore, a comparative analysis is carried out to determine how the performance of the bat algorithm (BA), Grey Wolf Optimizer (GWO), and butterfly optimization algorithm (BOA) is affected by population size, total iterations, and ten initialization techniques.

Akay et al.^32^ presented an extensive study of metaheuristic methods used in deep neural network (DNN) design. Various DNN structures, optimization issues, encoding approaches, evolutionary operators, techniques for validation, and datasets are all covered. The study addresses the benefits, drawbacks, and potential directions of metaheuristic techniques by classifying them according to the type of problem. Table 2. summarizes the most recent research articles that discuss optimization techniques.

**Table 2:** Complete summary of the literature review of recent research articles in state of art of optimization techniques.

Authors	Algorithm/Model	Strengths	Challenges
Rajwar et al.^21^	540 metaheuristic architectures (MAs)	Thorough assessment and classification based on control parameters are useful for novices	Proliferation of MAs, doubts on the uniqueness of optimization strategies
Abd Elaziz et al.^22^	Swarm intelligence (SI) and evolutionary computing (EC) for DNN optimization	Improves the accuracy of DNN and optimizes hyperparameters and structures for big datasets	Complexity of optimization in large-scale applications
Dey et al.^23^	Golden Ratio-based Equilibrium Optimization (GREO) algorithm	High accuracy in emotion classification using speech analysis, and efficient feature selection	Computational cost and complexity
Myriam et al.^24^	Hybrid Particle Swarm Optimization (PSO) and Al-Biruni Earth Radius (BER) Optimization	High accuracy in oral cavity cancer detection, and stability confirmed by statistical tests	Scalability to other medical conditions
Zhang et al.^25^	Hybrid AOAAO (Arithmetic Optimization + Aquila Optimizer)	Efficient performance, rapid convergence, and high precision in engineering problems	Managing parameter unpredictability
Sohlot et al.^26^	Hybrid OBHS (Opposition-based Harmony Search) and MRFO (Manta Ray Foraging Optimization)	High precision in fertilizer optimization, and notable environmental impact reduction	Adaptability to different agricultural scenarios
Baghdadi et al.^27^	MRFO optimization and transfer learning for CNN	High accuracy in breast cancer diagnosis, effective in ultrasound and histology data classification	Integration with real-time diagnostic systems
Wang et al.^28^	IHAOHHO (Aquila Optimizer + Harris Hawks Optimizer)	Improved exploration and extraction capabilities, better performance on technical issues and benchmark measures	Complexity in combining multiple optimization techniques
Nguyen et al.^29^	Combination of HHO, SSA, MSA, BSA, GOA, and PSO with Xgboost (XGR)	High precision in soil salinity monitoring, outperforms traditional models	Implementation in different geographical regions and conditions
Wen et al.^30^	Enhanced Remora Optimization Algorithm (IROA) with Lens Opposition-Based Learning (LOBL)	Escapes local optima efficiently and outperforms existing meta-heuristic approaches in benchmark functions and automotive design	Complexity in optical lens imaging-based optimization techniques
Agushaka et al.^31^	Comparative analysis of initialization strategies for BA, GWO, and BOA	Assesses the efficacy of different initialization strategies provides insights for further investigation	Dependence on population size and total iterations
Akay et al.^32^	Metaheuristic methods for DNN design	A comprehensive study on DNN structures, optimization issues, encoding approaches, evolutionary operators, validation techniques, and datasets	Complexity in balancing benefits and drawbacks of various metaheuristic techniques for different types of problems

According to Tables 1 and 2, the Enhanced Object Detection model, which solves specific constraints while building on the strengths of previous systems, makes substantial contributions to the field of remote sensing image analysis. Unlike prior models, which frequently focus on specific applications or struggle to optimize object detection algorithms, this improved technique makes use of metaheuristic and hybrid metaheuristic optimizers, which were used in YOLOv7 and YOLOv8, respectively. The combination of these optimizers enables consistent performance across varied datasets by successfully handling imbalanced data and boosting detection accuracy. The study extends existing approaches by exploiting the strengths of metaheuristic and hybrid metaheuristic optimizers, particularly YOLOv7 and YOLOv8, to ensure consistent performance across diverse datasets. This proposed approach improves the capacity for identifying complex details in remotely sensed images, establishing a new standard for object detection precision in the field.

## Materials and methods

**Fig. 1:**
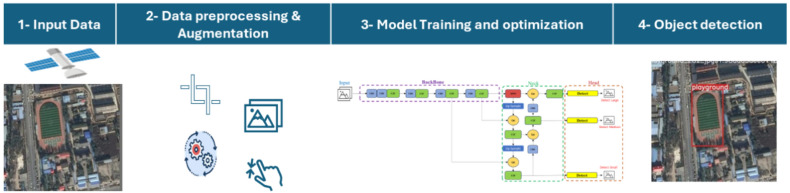
The main framework of the proposed object detection.

This study proposed different metaheuristic optimization algorithms with YOLOv7 and YOLOv8 to enhance the performance achieved in object detection from remote sensing imagery. The suggested framework offers a useful approach for improving detection precision without necessitating the development of new structured algorithms. To ensure the effectiveness and robustness of the methodology, extensive testing is conducted across various remote-sensing image datasets. This section discusses the suggested framework’s typical procedures including the main three steps: (i) data preprocessing and augmentation, (ii) metaheuristic optimization & YOLO models training, and (iii) image detection, which are represented in Fig. 1. The following subsections illustrate the utilized optimization algorithm, detection algorithms, and dataset.

### Datasets

#### Datasets description

Remote sensing datasets have been assigned for a variety of applications and purposes, including classification, object detection, and segmentation. This article is specifically utilized for object detection of datasets with multiple classes, including the Remote Sensing Object Detection (RSOD), and the Northwestern Polytechnical University Very High Resolution 10 (NWPU VHR-10) Datasets. To ensure that the proposed model can be utilized successfully for all detection purposes, the two datasets are further distinguished based on the types of detection classes and the number of applications. Table 3 describes the utilized two dataset’s main parameters.

**Table 3:** The parameters of the datasets.

Dataset	No. of classes	No. of labeled images	No. of object labels	Image size	Images type	Application
RSOD	4	976	6950	640 × 640 × 3	JPG	Object Detection
NWPU VHR-10	10	650	3775	640 × 640 × 3	JPG	Object Detection

##### RSOD dataset

**Fig. 2:**
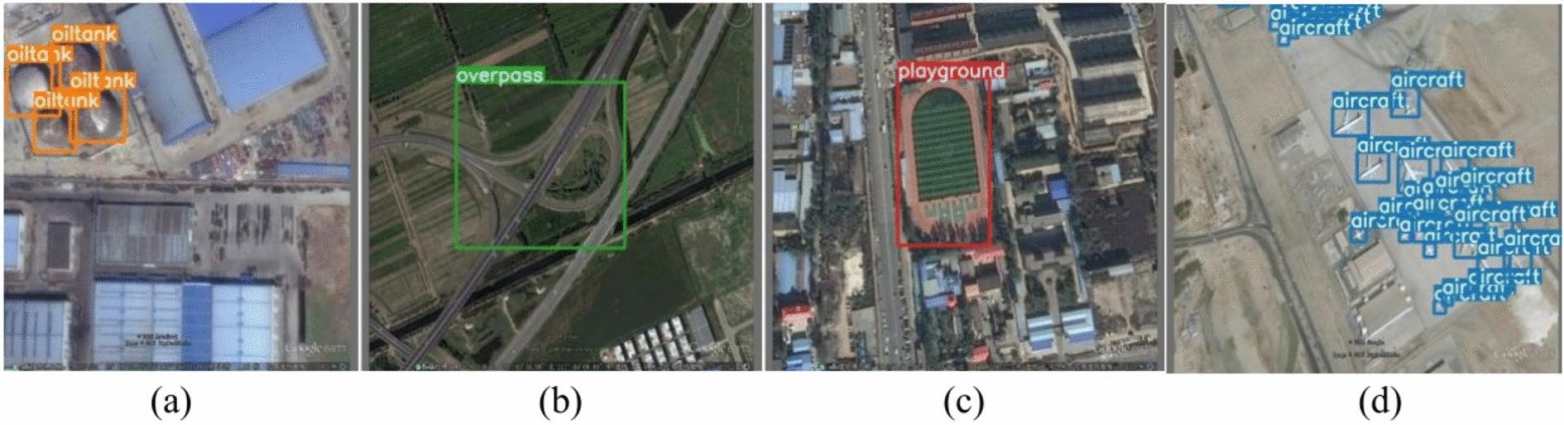
The RSOD dataset sample images: (**a**) sample image contains oil tanks, (**b**) sample image contains overpass, (**c**) sample image contains playground, (**d**) sample image contains aircrafts.

The RSOD dataset consists of 976 images sourced from Google Earth and Tianditu, featuring spatial resolutions ranging from 0.3 to 3 m. It includes a total of 6950 object labels, with the following breakdown: 4993 airplanes, 1586 oil tanks, 191 playgrounds, and 180 overpasses^33,34^. Figure 2 represents RSOD dataset sample images with their corresponding boundary boxes.

##### NWPU VHR-10 dataset

**Fig. 3:**
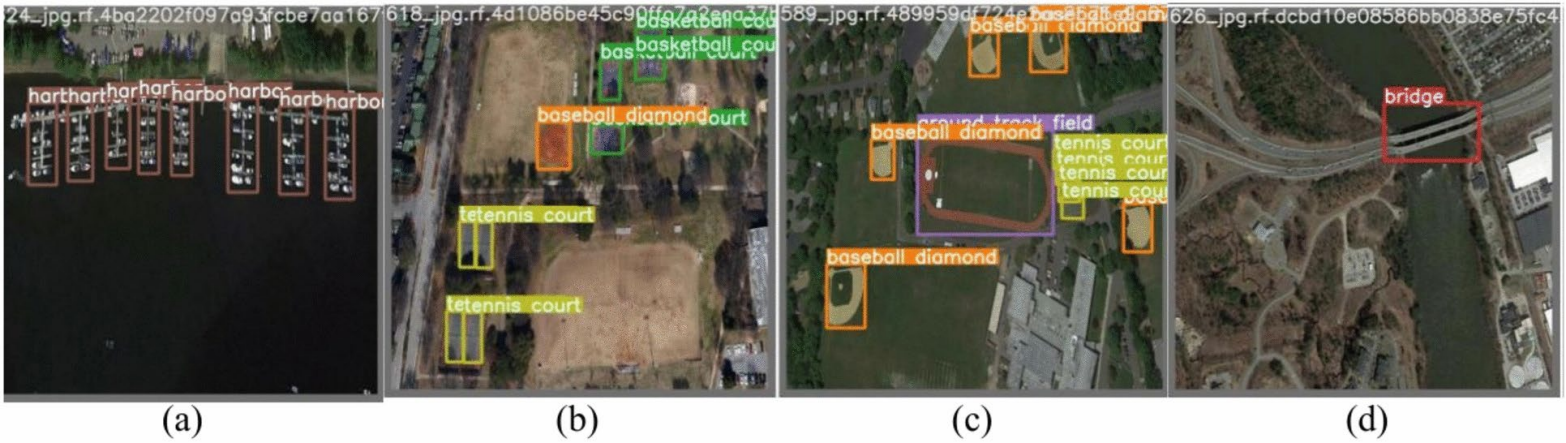
The NWPU VHR-10 dataset sample images: (**a**) sample image contains harbors, (**b**) sample image contains tennis court, baseball diamond, and basketball courts, (**c**) sample image contains ground track, baseball diamonds, and tennis courts, (**d**) sample image contains bridge.

The NWPU VHR-10 is a ten-class remote sensing objects detection dataset. It consists of 650 remote sensing pictures collected from Google Earth, with spatial resolutions varying from 0.5 to 2 m. The NWPU VHR-10 dataset includes thorough annotations for multiple classes, which provide ground truth for object detection. These classes include 757 planes, 655 storage tanks, 524 tennis courts, 477 vehicles, 390 baseball diamonds, 302 ships, 224 harbors, 159 basketball courts, 163 ground track fields, and 124 bridges^35,36^. Figure 3 represents NWPU VHR-10 dataset sample images with their corresponding boundary boxes.

#### Datasets preprocessing

The article presents a preprocessing pipeline designed exclusively for YOLOv7 and YOLOv8 object identification. It is primarily concerned with two essential aspects: making sure that all the images are scaled to 640 × 640 × 3 and reformatting label files to meet the YOLOv7 and YOLOv8 formats, which ensures compatibility and standardization for best performance^37^. The preprocessing step’s initial procedure was to open the annotation files that accompanied the tagged photos, which contained significant details such as the object’s class labels and bounding box coordinates. The images were then resized to a size of 640 × 640 pixels to guarantee consistency throughout the collection. To better depict the object’s position and size, the bounding box coordinates were normalized by dividing them by the scaled image’s width and height. The annotation data for each object was then formatted in the YOLO format, with the class label followed by the bounding box’s normalized coordinates (center x and y coordinates, width, and height). Finally, the formatted annotations were saved in text files, with each line corresponding to an object in the image.

#### Datasets augmentation

Image augmentation is a crucial approach in computer vision, especially for object detection purposes. It entails creating new training samples from present information to increase the variety and amount of the dataset^38^. This method is critical for deep learning models because it prevents overfitting and ensures that the model obtains attributes that are invariant under specific modifications. Models can better generalize to new, previously untrained data by incorporating variables such as rotations, scaling, flipping, and color alterations^39^. Different augmentation techniques have been utilized in this article including:

##### Geometric transformations

Geometric transformations include cropping, flipping, rotating, and scaling. These modifications change the spatial arrangement of pixels in the picture, allowing the model to learn spatial invariance. The article utilized flipping a picture horizontally or vertically (with a probability of 0.5).

##### Color space transformations

Another successful enhancement approach is to adjust the image’s color space. Adjusting the image’s hue, saturation, and value (HSV) imitates various lighting conditions and camera settings. The article utilized hue, saturation, and value (HSV) augmentations by values of 0.015, 0.7, and 0.4 respectively provide the percentage by which the hue, saturation, and value should be changed, respectively. These modifications allow the model to retain performance even when the color conditions in the input data change.

##### Mixing techniques

Mixing pictures is a more complex augmentation approach that combines several photos to generate fresh training examples. The mosaic and mix-up parameters, with a probability of 0.5 and 0.15, are applied during the augmentation stage in the article. Mix-up mixes information from numerous photos, resulting in a more robust model capable of handling overlapping objects and settings.

##### Translation and scaling

Translation and scaling are used to create the appearance of things being at varied distances and locations in the photographs. With the applied 0.1 translation parameter, the picture can be displaced by up to 10% of its overall width or height in any direction. Furthermore, with an applied scaling value of 0.5, the image’s size is adjusted by a particular amount, which can imitate zooming in or out.

**Fig. 4:**
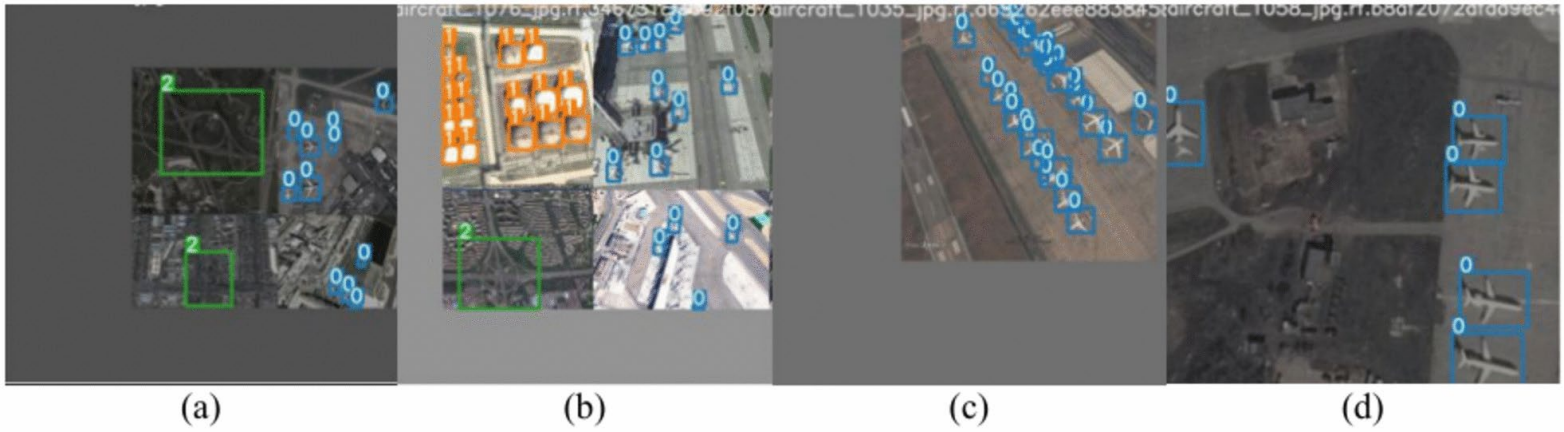
The results of the augmentation process sample images: (**a**) sample image shows the results of mixing technique with scaling technique, (**b**) sample image shows the results of mixing technique with scaling technique, (**c**) sample image shows the results of scaling technique, (**d**) sample image shows the results of flipping technique with color space transformation.

The results of the augmentation process sample images are represented in Fig. 4.

### Optimization algorithms

Metaheuristic optimization methods, such as Particle Swarm Optimization (PSO), Genetic Algorithm (GA), Grey Wolf Optimization (GWO), Aquila Optimizer (AO), Remora Optimization Algorithm (ROA), and hybrid PSO and GWO (HPSGWO) are used to optimize the hyperparameters of the YOLOv7 and YOLOv8 models. These optimization techniques were motivated by their demonstrated success in optimizing difficult, multidimensional challenges. PSO is noted for its fast convergence, making it perfect for quickly recognizing near-optimal solutions in huge search spaces. GA’s genetic operators provide extensive search capabilities, allowing for the exploration of a wide range of solution spaces. GWO maintains a balance between exploration and exploitation, which is critical for avoiding local optima. AO’s adaptive techniques enable it to successfully navigate dynamic landscapes, but ROA’s new symbiosis-inspired approach provides a distinct perspective on optimization. The hybrid HPSGWO combines the strengths of PSO and GWO to improve overall performance. These algorithms iteratively explore the solution space to determine the best configuration for the models, maximizing detection precision while minimizing computing costs. The proposed system uses metaheuristic optimization to adapt models to the specific characteristics of the target application, eliminating the need for manual tuning or algorithm modification. In the field of remote sensing and object detection, the application of optimization algorithms is an effective method for optimizing parameters inherent in algorithms such as You Only Look Once (YOLO). Researchers hope to improve YOLO’s effectiveness by adjusting parameters such as anchor box sizes, aspect ratios, and confidence thresholds.

### Particle swarm optimization (PSO)

Particle Swarm Optimization (PSO) is a popular metaheuristic optimization approach based on social behaviors, namely the collective movement of bird flocks or fish schools. Its application extends into different sectors, including remote sensing. PSO uses a population of potential solutions, known as particles, each particle has its unique position and velocity to navigate a multidimensional search space. The essential assumption is that these particles will iteratively perfect themselves towards the global optimum, aided by their individual and collective experiences^40^. The mathematical model of PSO can be formed by updating the velocity quantity using Eq. (1) and the position using Eq. (2).^41^

1 $${\vartheta }_{i,d}\left(t+1\right)= \omega \times {\vartheta }_{i,d}\left(t\right)+ {c}_{1}\times {r}_{1}\times \left({{P}_{b}}_{i,d}-{P}_{i,d}\left(t\right)\right)+{c}_{2}\times {r}_{2}\times \left({{G}_{b}}_{d}-{P}_{i,d}\left(t\right)\right)$$

2 $${P}_{i,d}\left(t+1\right)={P}_{i,d}\left(t\right)+ {\vartheta }_{i,d}\left(t+1\right)$$

where, $\omega$ is the inertia weight for global and local exploration balancing, ${c}_{1}$ and ${c}_{2}$ are the acceleration constants, ${r}_{1}$ and ${r}_{2}$ are random numbers in the range (0,1), ${\vartheta }_{i,d}$ is the initial velocity, ${P}_{i,d}$ is the initial position for each i which is the particle and d which is the dimension, ${P}_{b}$ is the agent’s best solution, and ${G}_{b}$ is the global best solution. Algorithm 1 presents the pseudocode of the PSO.

**Algorithm 1:**
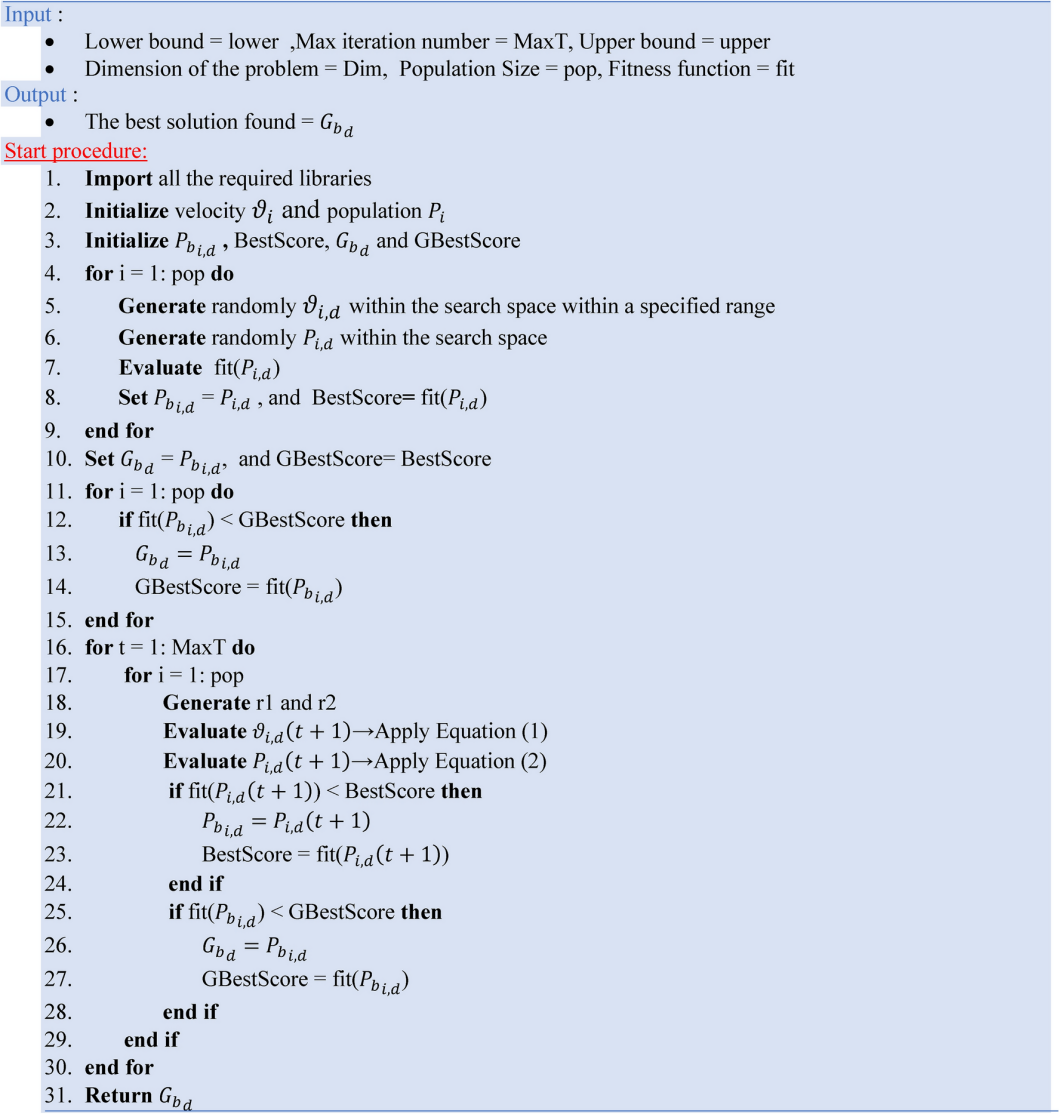
The pseudocode of the PSO.

### Genetic algorithm (GA)

Genetic Algorithm (GA) is a powerful optimization method inspired by the principles of natural selection and genetics, with excellent possibilities for improving object detection in satellite imaging applications. GA models evolutionary processes to rapidly explore complex solution spaces^42^. The algorithm starts with an initial population of potential solutions, which are assessed using an objective function that represents their fitness. Chromosome representation, selection, crossover, mutation, and the computing of fitness functions are all important aspects of GA. The GA method starts by initializing a population of n chromosomes. The fitness of each chromosome is calculated, and two chromosomes are chosen depending on their fitness scores. To create children, a single-point crossover operator is applied to these selected chromosomes. This offspring is then subjected to a uniform mutation process, resulting in the production of new offspring. This new offspring is introduced into the new population. The selection, crossover, and mutation operations are carried out until the new population is complete. GA dynamically modifies its search strategy, allowing it to analyze various individuals and generate multiple best solutions. It maintains population variety by updating the original schema with an updated version. The following Eqs. (3–5) represent the mathematical representation of the main aspects of GA^43^.

3 $${S}_{p}\left(x\right)=\frac{f(x)}{\sum_{i=1}^{N}f(x)}$$

where ${S}_{p}$ is the selection probability, $f(x)$ is the fitness function, $x$ is the individual solution, and $N$ is the solution size.

4 $${x}_{offspring}=concat({x}_{\text{1,1}:k}, {x}_{2,k+1:N})$$

where, $N$ is the length of the solution, $k$ is the point of the cross-over.

5 $$x_{mutated} = \left\{ {\begin{array}{*{20}c} {x_{offspring} } & {with \;probaility \;1 - p_{m} } \\ {mutate\left( {x_{offspring} } \right)} & {with \;probaility \;p_{m} } \\ \end{array} } \right.$$

where, ${x}_{offspring}$ is the output of the cross-over operation or the offspring, and ${p}_{m}$ probability parameter set before running the genetic algorithm. Algorithm 2 presents the pseudocode of the GA.

**Algorithm 2:**
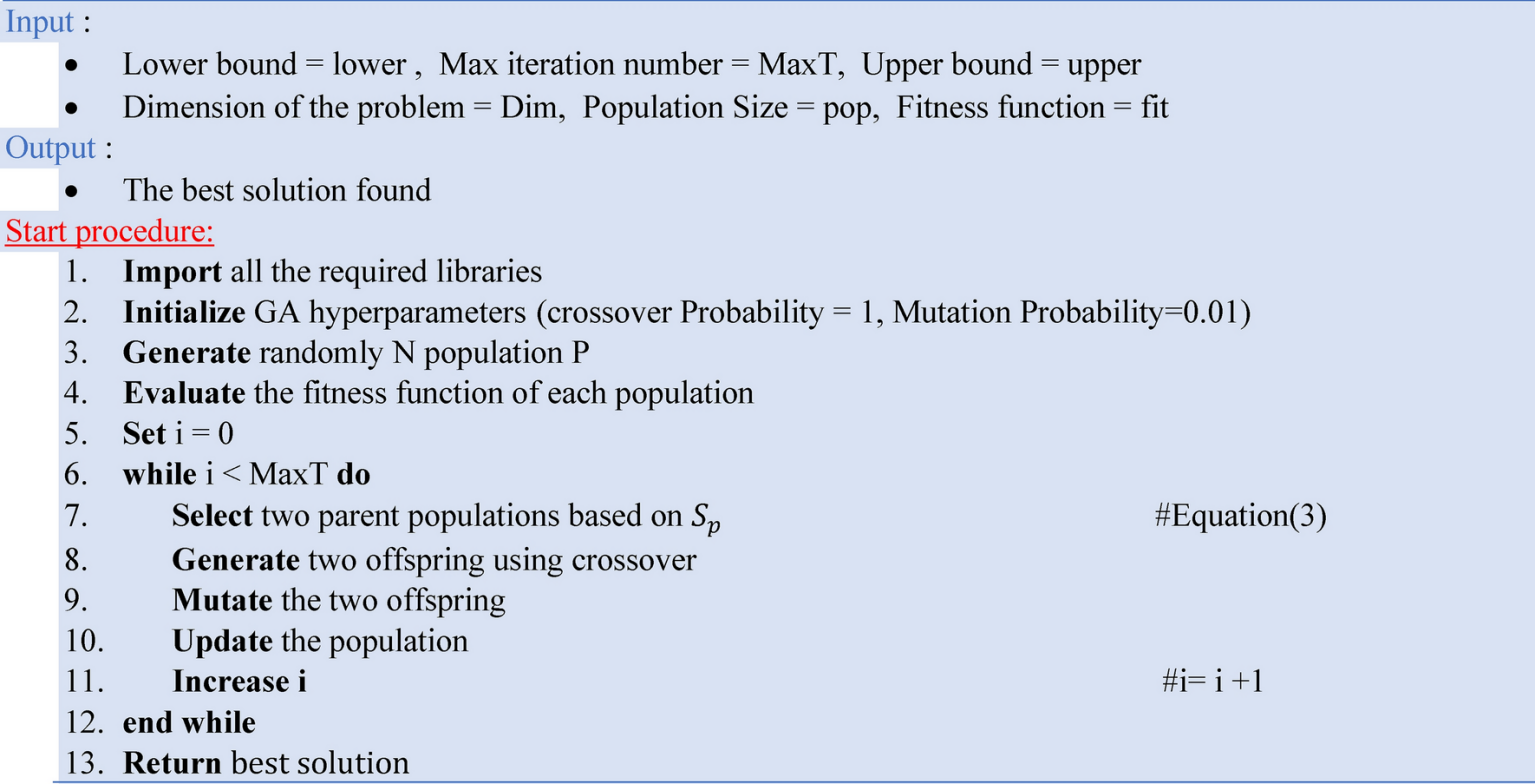
The pseudocode of the GA..

### Gray wolf optimizer (GWO)

The Grey Wolf Optimizer (GWO) is a population-based metaheuristic optimization algorithm inspired by grey wolves’ social hierarchy and hunting behavior. It uses the leadership hierarchy of wolves to repeatedly search for the best solutions within complicated searching areas. In the field of remote sensing, GWO presents a promising strategy for optimizing parameters and settings in object detection algorithms, improving detection accuracy and efficiency. The GWO has three major steps: initialization, updating, and selection. During the initialization step, a random set of solutions is generated within the search space, with each solution representing a wolf in the pack. A grey wolf’s mathematical location (or solution) can be described as ${x}_{i}=({x}_{i1},{x}_{i2}, {x}_{i3},\dots ,{x}_{iD})$, where i = 1,2, …, N (N is the total number of wolves or population size) and D is the problem dimension^44^. The prey is the optimum we must seek where $\alpha$ is the Fitness Solution, $\beta$ represents the second-best solution, $\delta$ the third-best solution, and ${\varvec{\omega}}$ are the rest of the solutions. The mathematical representation of the grey wolf social hierarchy is outlined by the following formula Eqs. (6–12).^45^ The initial step occurs when grey wolves surround prey^45^.

6 $$\overrightarrow{D}=\left|\overrightarrow{C}\cdot \overrightarrow{{P}_{t}}\left(i\right)- \overrightarrow{P}(i)\right|$$

7 $$\overrightarrow{P}\left(i+1\right)=\overrightarrow{{P}_{t}}\left(i\right)-\overrightarrow{D}\cdot \overrightarrow{A}$$

where, $\overrightarrow{C}$ and $\overrightarrow{A}$ are vectors of coefficients, $\overrightarrow{P}$ is the gray wolf position vector, ${P}_{t}$ is the position of the target prey, and $i$ is the iteration number. $\overrightarrow{C}$ and $\overrightarrow{A}$ can be described mathematically as follows^45^:

8 $$\vec{A} = 2\vec{a} \cdot \overrightarrow {{r_{1} }} - \vec{a},\;\;\vec{a} = 2\left( {\frac{1 - i}{I}} \right)$$

9 $$\overrightarrow{C}=2\cdot \overrightarrow{{r}_{2}}$$

where $\overrightarrow{a}$ Linearly reduced between two and zero over the iterations, $i$ is the iteration number, I is the maximum number of iterations and $\overrightarrow{{r}_{1}}$ and $\overrightarrow{{r}_{2}}$ are random vectors with values between zero and one.

10 $$\overrightarrow {{D_{\alpha } }} = \left| {\overrightarrow {{C_{1} }} \cdot \overrightarrow {{P_{\alpha } }} - \vec{P}} \right|,\;\overrightarrow {{D_{\beta } }} = \left| {\overrightarrow {{C_{2} }} \cdot \overrightarrow {{P_{\beta } }} - \vec{P}} \right|,\;\overrightarrow {{D_{\delta } }} = \left| {\overrightarrow {{C_{3} }} \cdot \overrightarrow {{P_{\delta } }} - \vec{P}} \right|$$

11 $$\overrightarrow {{P_{1} }} = \overrightarrow {{P_{\alpha } }} - \overrightarrow {{A_{1} }} \cdot \overrightarrow {{D_{\alpha } }} ,\;\overrightarrow {{P_{2} }} = \overrightarrow {{P_{\beta } }} - \overrightarrow {{A_{2} }} \cdot \overrightarrow {{D_{\beta } }} ,\;\overrightarrow {{P_{3} }} = \overrightarrow {{P_{\delta } }} - \overrightarrow {{A_{3} }} \cdot \overrightarrow {{D_{\delta } }}$$

12 $$\vec{P}\left( {t + 1} \right) = \frac{{\overrightarrow {{P_{1} }} + \overrightarrow {{P_{2} }} + \overrightarrow {{P_{3} }} }}{3}$$

**Fig. 5:**
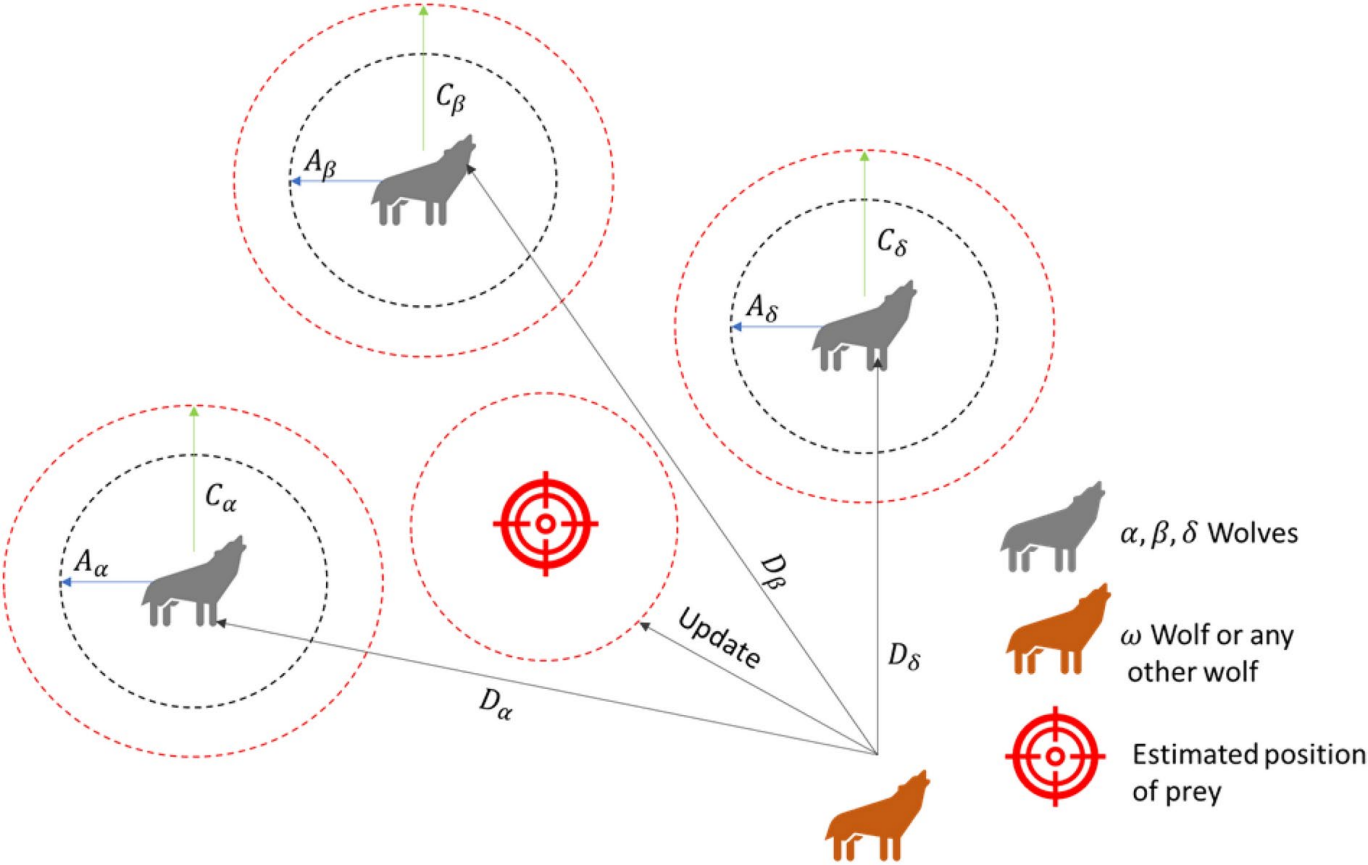
The Gray Wolf’s behavior of hunting.

Grey wolves hunt for prey based on the positions of their leaders (alpha, beta, and delta), diverging from one another. Algorithm 3 presents the pseudocode of GWO. Figure 5 represents the multiple options for catching prey.

**Algorithm 3:**
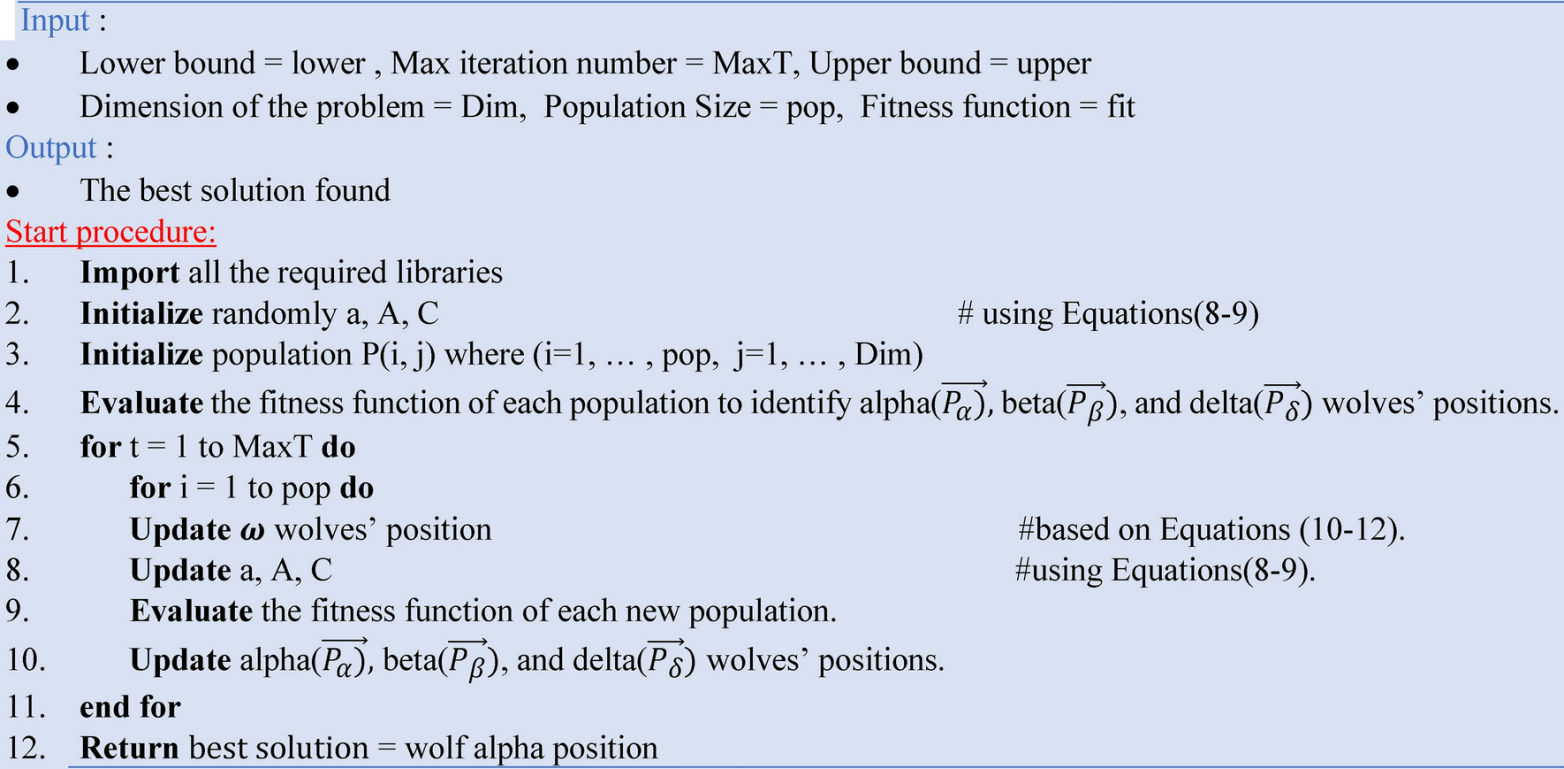
The pseudocode of the GWO.

### Aquila optimizer (AO)

The Aquila Optimizer (AO) is a contemporary bio-inspired metaheuristic algorithm based on the hunting behavior of the eagle, Aquila Chrysaetos. The traditional AO emphasizes five key steps: initialization, expanded exploration, narrow exploration, expanded exploitation, and narrow exploitation. During the initialization step, a random set of solutions is created from the search space. Each solution symbolizes an eagle in the flock, defined as ${x}_{i}=({x}_{i1},{x}_{i2}, {x}_{i3},\dots ,{x}_{iD})$, where i = 1,2, …, N (N is the population size), and D is the problem dimension^46^. The eagles’ hunting behavior is then recreated throughout the hunting phase. Multiple equations are used to mathematically model this behavior. The eagles’ positions are updated based on the position of the best eagle (those having the highest fitness) and the population’s indicated position. The second step expanded exploration equations can be represented as follows in Eqs. (13–14).^47,48^

13 $${P}_{1}\left(i+1\right)={P}_{b}(i)\times \left(1-\frac{i}{I}\right)+\left({P}_{m}\left(i\right)-{P}_{b}\left(i\right)*random\right)$$

where, ${P}_{1}\left(i+1\right)$ the next iteration solution, ${P}_{b}(i)$ is the best solution found till the current iteration ($i$), $I$ is the number of iteration maximum values, $random$ represents a random number between [0,1], and ${P}_{m}\left(i\right)$ is the mean value of the location of the current solution^47,48^.

14 $${P}_{m}\left(i\right)= \frac{1}{S}\sum_{n=1}^{S}{P}_{n}\left(i\right), {\forall }_{m}=\text{1,2}, \dots ,d$$

where d presents the dimension, S presents the size of the population, and d presents the length of the search space.

The third step narrow exploration, the second hunting technique, equations can be represented as follows in Eqs. (15–22).^47,48^

15 $${P}_{2}\left(i+1\right)={P}_{b}(i)\times Levy\left(d\right)+\left({P}_{r}\left(i\right)-\left(a-b\right)*random\right)$$

where, ${P}_{2}\left(i+1\right)$ the next iteration solution, ${P}_{b}(i)$ is the best solution found till the current iteration ($i$), ${P}_{r}\left(i\right)$ is a random number between [1, S], $random$ represents a random number between [0,1], and a and b represent the spiral shape in the search and are calculated as follows in Eqs. (16–20).

16 $$a=r \times \text{sin}(\varnothing )$$

17 $$b=r \times \text{cos}(\varnothing )$$

18 $$r= {r}_{1}+U \times {d}_{1}$$

19 $$\varnothing = -\varepsilon \times {d}_{1}+{\varnothing }_{1}$$

20 $${\varnothing }_{1}=\frac{3\times \pi }{2}$$

where ${r}_{1}$ is a value in the range of (1, 20) fixed for each search cycle, $U$ is constant and equal to 0.00565, ${d}_{1}$ is an integer value in the range of (1,d), and $\varepsilon$ is constant and equal to 0.005.

21 $$Levy\left(d\right)=c \times \frac{v \times \alpha }{{\left|u\right|}^{\frac{1}{\beta }}}$$

where c is a constant and equal to 0.01, $\beta$ is constant and equal to 1.5, v and u are numbers randomly picked in the range of [0,1], and $\alpha$ can be calculated by applying the following Eq. (22).22

The 4th stage, Expanded Exploitation, is based on the third hunting mechanism and involves low flying with a steady descending attack. Aquila moves nearer to the target before attacking. Equation (23) appropriately describes the effect of Aquila^47,48^.

23 $${P}_{3}\left(i+1\right)={(P}_{b}(i)\times {P}_{m}\left(i\right))\times \sigma -random+((upper-lower) \times random+ lower) \times \mu$$

where, $${P}_{3}\left(i+1\right)$$ the next iteration solution, $${P}_{b}(i)$$ is the best solution found till the current iteration ($$i$$), $${P}_{m}\left(i\right)$$ is the mean value of the location of the current solution, $$random$$ represents a random number between [0,1], $$upper and lower$$ donates the upper bound and the lower bound of the given problem respectively, and $$\sigma$$ and $$\mu$$ are Fixed exploitation modification settings.

In the last phase, the notion is based on Aquila’s walk-and-grab attack to create Narrowed Exploitation, which is statistically represented in Eq. (24).

24 $${P}_{4}\left(i+1\right)={P}_{b}\left(i\right)\times FQ-\left({O}_{1}\times P\left(i\right)\times random\right)-{O}_{2}\times Levy\left(d\right)+random\times {O}_{1}$$

where, ${P}_{3}\left(i+1\right)$ the next iteration solution, ${P}_{b}(i)$ is the best solution found till the current iteration ($i$), $P\left(i\right)$ is the current solution of the ith iteration, and $FQ$ is the function of the quality Used to balance the search techniques. $FQ$, various motions ${O}_{1}$, and fight slope ${O}_{2}$ can be calculated using Eqs. (25–27).^47,48^

25 $$FQ\left(i\right)={i}^{\frac{2\times random-1}{{(1-I)}^{2}}}$$

26 $${O}_{1}=2\times random-1$$

27 $${O}_{2}=2\times (1-\frac{i}{I})$$

where i is the iteration number, I is the iteration maximum number, and random is a random value between 0 and 1. Algorithm 4 presents the pseudocode of the AO.

**Algorithm 4:**
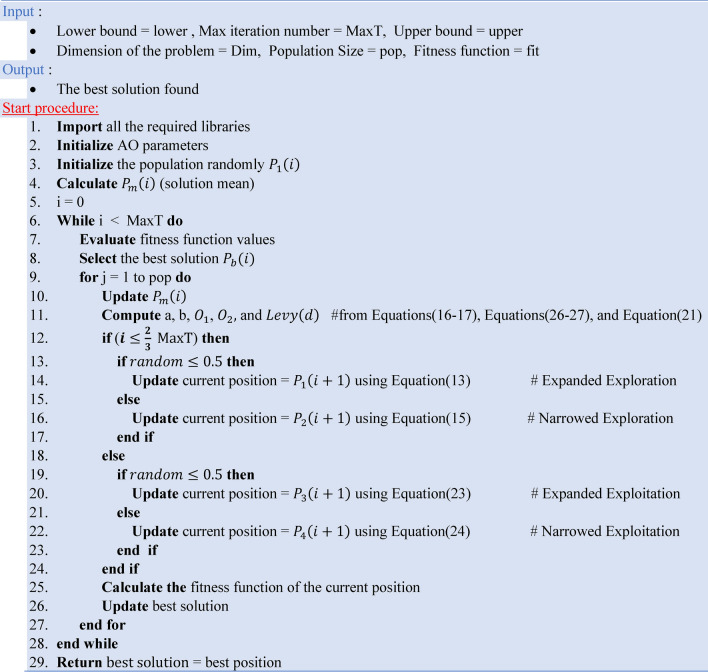
The pseudocode of the AO.

### Remora optimization algorithm (ROA)

The Remora Optimization Algorithm (ROA) is inspired by the hunting behavior of remora fish. These fish have parasitic relationships with bigger hosts, such as swordfish and whales, and rely on them for nourishment. The ROA algorithm uses the ideas of Swordfish Optimization (SFO) and Whale Optimization Algorithm (WOA) to represent the exploration and exploitation stages, respectively^49^. To optimize the search method, the algorithm incorporates empirical assaults and host feeding between multiple hosts. To begin the optimization process, a collection of random solutions is produced in the domain of search, which will serve as the first ROA solutions^50^. The remora fish’s location is computed using the following Eq. (28).^49,50^

28 $${P}_{i}=lower+random\times (upper-lower)$$

where, ${P}_{i}$ is the remora fish position, lower is the search space lower bound, upper is the search space upper bound, and $random$ represents a random number between [0,1].

During the exploration stage, the remora fish hooks itself to the flag fish and travels beside it. The SFO algorithm provides the fish’s position update formula, which can be represented in Eq. (29).

29 $${P}_{n}^{i+1}= {P}_{b}^{i}-\left(random\times \left(\frac{{P}_{b}^{i}+{P}_{random}^{i}}{2}\right)-{P}_{random}^{i}\right)$$

where, ${P}_{n}^{i+1}$ the next iteration solution, ${P}_{b}^{i}$ is the current best solution in the i^th^ iteration and ${P}_{random}^{i}$ represents the random remora currently.

During the exploratory stage, the remora fish launches a limited number of exploratory assaults dependent on the position of the host and the position of the previous generation of remora. The modified equation for the remora’s location is presented in Eq. (30).^49,50^

30 $${P}_{ta}={P}_{n}^{i}+\left({P}_{n}^{i}+{P}_{pg}\right)\times random$$

where, ${P}_{ta}$ presents the fish’s tentative assault, ${P}_{pg}$ represents the location of the fish’s previous generation, and $random$ represents a random number between [0,1]. The host switch method depends on the following Eqs. (31–32).^49,50^

31 $$fit\left({P}_{n}^{i}\right)<fit\left({P}_{ta}\right)$$

32 $$C\left(n\right)=round(random)$$

where, $fit\left({P}_{n}^{i}\right)$ and $fit\left({P}_{ta}\right)$ denote the fitness values of ${P}_{n}^{i}$ and ${P}_{ta}$ respectively, $random$ represents a random number between [0,1], and $C\left(n\right)$ is a variable that determines the host of the remora parasite, having a starting value of 0 or 1 (If $C\left(n\right)$ equals 0, the remora will be hosted on the whale; if $C\left(n\right)$ equals 1, the remora will be housed on the swordfish).

During the exploitation stage, the remora fish hooks itself to the whale and travels alongside it. The WOA algorithm provides the location update formula for the remora, which is described in Eqs. (33–36).^49,50^

33 $${P}_{n}^{i+1}= \rho \times {e}^{\gamma }\times \text{cos}\left(2\pi \alpha \right)+{P}_{n}^{i}$$

34 $$\rho = \left|{P}_{b}^{i}{-P}_{n}^{i}\right|$$

35 $$\gamma =random \times \left(\alpha -1\right)+1$$

36 $$\alpha =-\left(1+\frac{i}{I}\right)$$

where, ${P}_{n}^{i+1}$ the next iteration solution, $\rho$ is the distance between the best solution position before updating and the current position, $\gamma$ is a value generated randomly in the range between [−1,1], $\alpha$ is the value which linearly decreased in the range between [−2,1], $random$ represents a random number between [0,1], i is the iteration number, and I is the number of maximum iterations.

When the remora fish approaches the host during the exploitation stage, it conducts a short-range search. The location update method for the remora’s location within this phase is represented in Eqs. (37–40).^49,50^

37 $${P}_{n}^{i+1}={P}_{n}^{i}+\tau$$

38 $$\tau =\beta \times \left({P}_{n}^{i}-\varepsilon \times {P}_{b}\right)$$

39 $$\beta =2\times \omega \times random- \omega$$

40 $$\omega =2 \times \left(1-\frac{i}{I}\right)$$

where, $\tau$ represents the distance moved by the remora, $\varepsilon$ is a fixed value and equal to 0.1, $\beta$ is the volume of the host fish, $\omega$ is the volume of the remora fish, $random$ represents a random number between [0,1], i is the iteration number, and I is the number of maximum iterations.

Algorithm 5 presents the pseudocode of the ROA.

**Algorithm 5:**
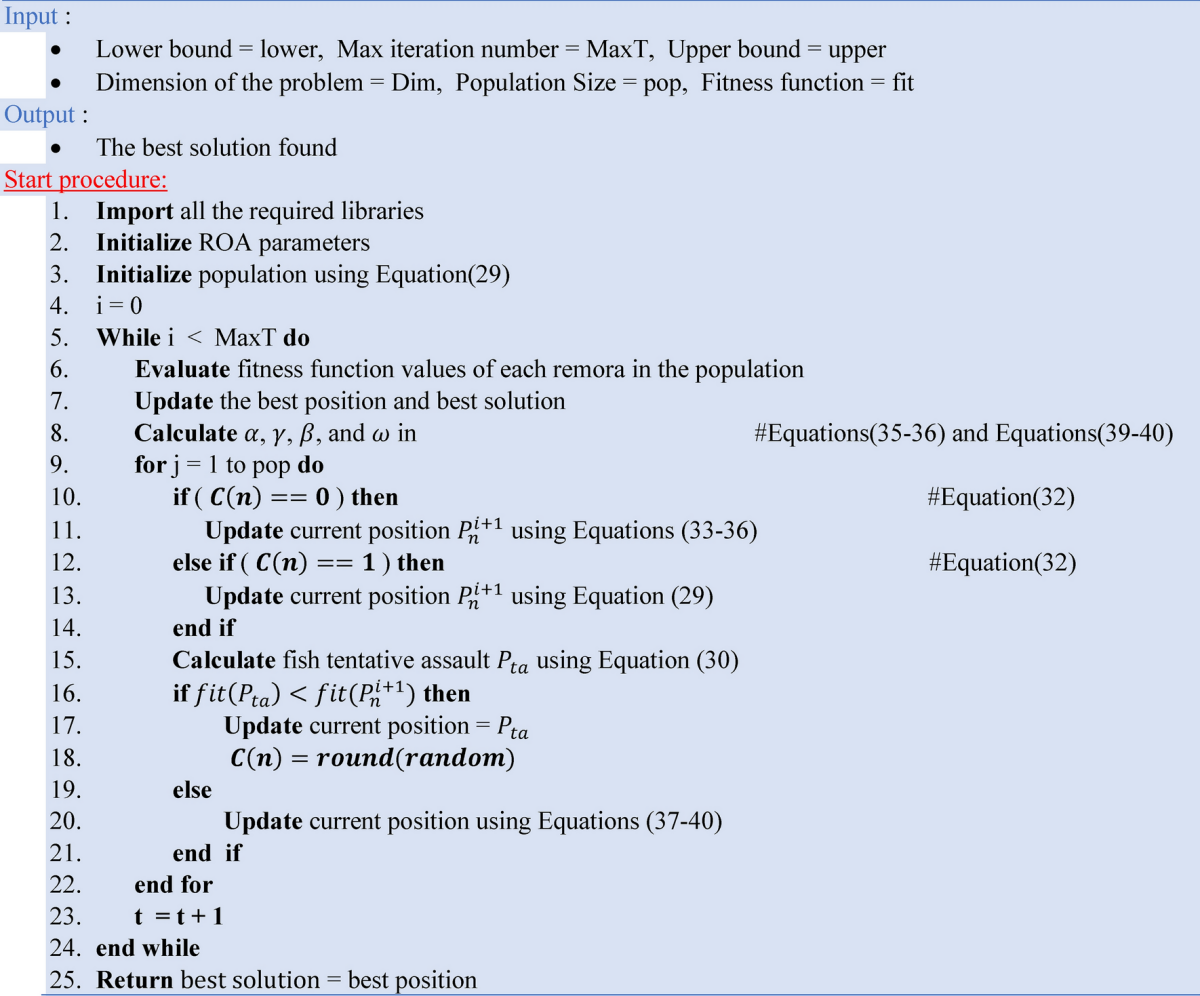
The pseudocode of the ROA.

### Hybrid PSO–GWO (HPSGWO)

**Fig. 6:**
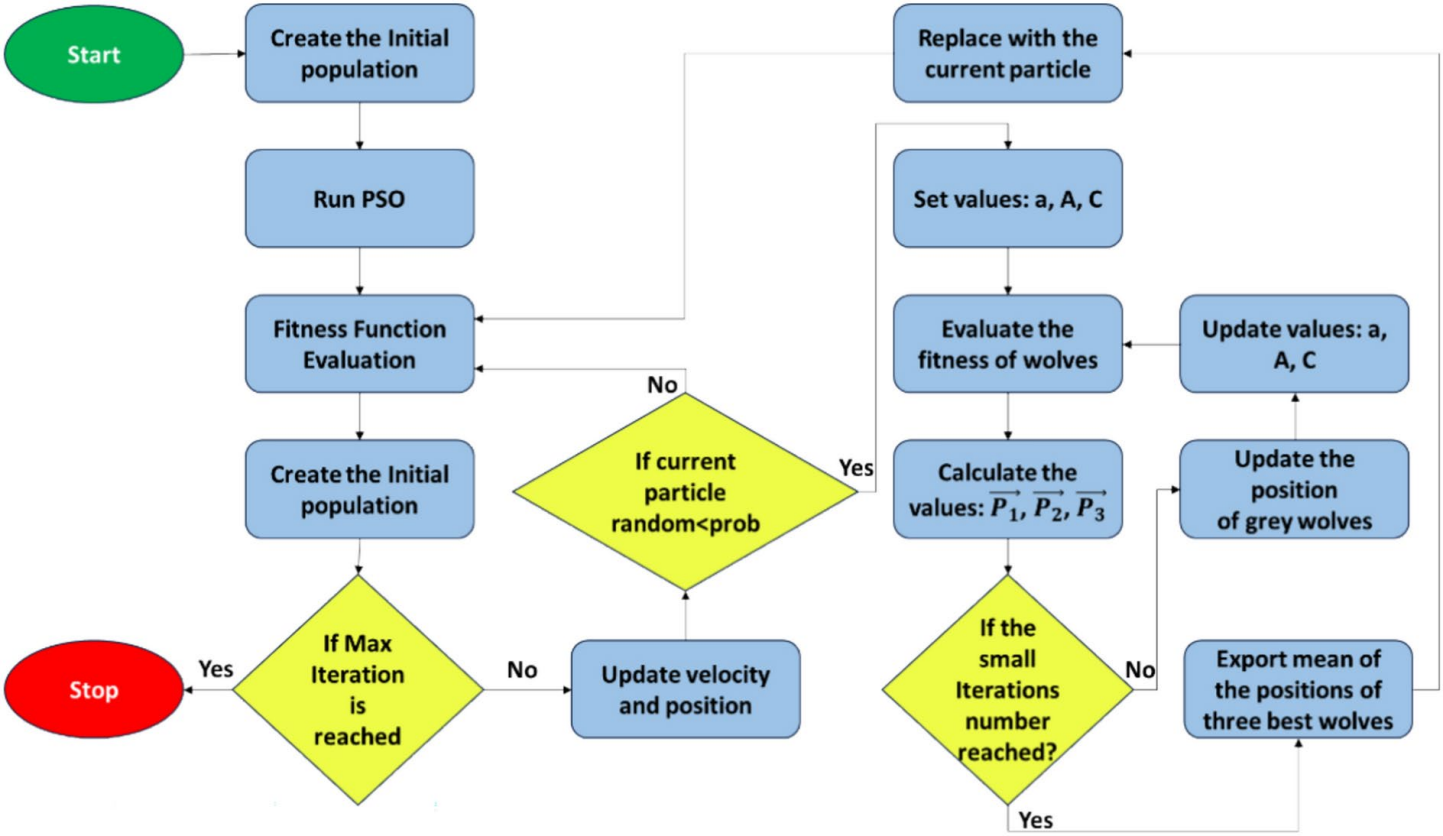
The Hybrid PSO–GWO Flow chart.

The Hybrid PSO-GWO method (HPSGWO) is used to address the issue of local minima trapping in optimization situations^51^. The HPSGWO method combines the Particle Swarm Optimization (PSO) and Grey Wolf Optimizer (GWO) algorithms, which improves PSO’s exploration capabilities. Specifically, GWO is used to direct a small number of particles to somewhat better locations, lowering the chance of early convergence to poor results^52^. This hybrid technique seeks to create a balance between exploration and exploitation by combining the benefits of both algorithms ^53^. Figure 6 represents the main flow chart of the Hybrid PSO–GWO algorithm steps and all the equations of PSO and GWO are predefined in sections "Optimization algorithms" (I and III).

### Object detection algorithms

YOLO (You Only Look Once) is a popular real-time object detection algorithm that uses a single neural network pass to predict both bounding boxes and class probabilities. It’s renowned for its speed and accuracy in detecting multiple objects within an image. Recent developments, such as YOLOv7 and YOLOv8, have been effectively employed in remote sensing object detection. Furthermore, these models have been used in hazard analysis, particularly in landslide detection, where rapid and precise identification of probable landslide regions is critical^54,55^. YOLO models’ capacity to quickly interpret large-scale remote sensing data and identify landslide-prone areas has made them useful tools in disaster management and environmental monitoring.

#### YOLOv7

**Fig. 7:**
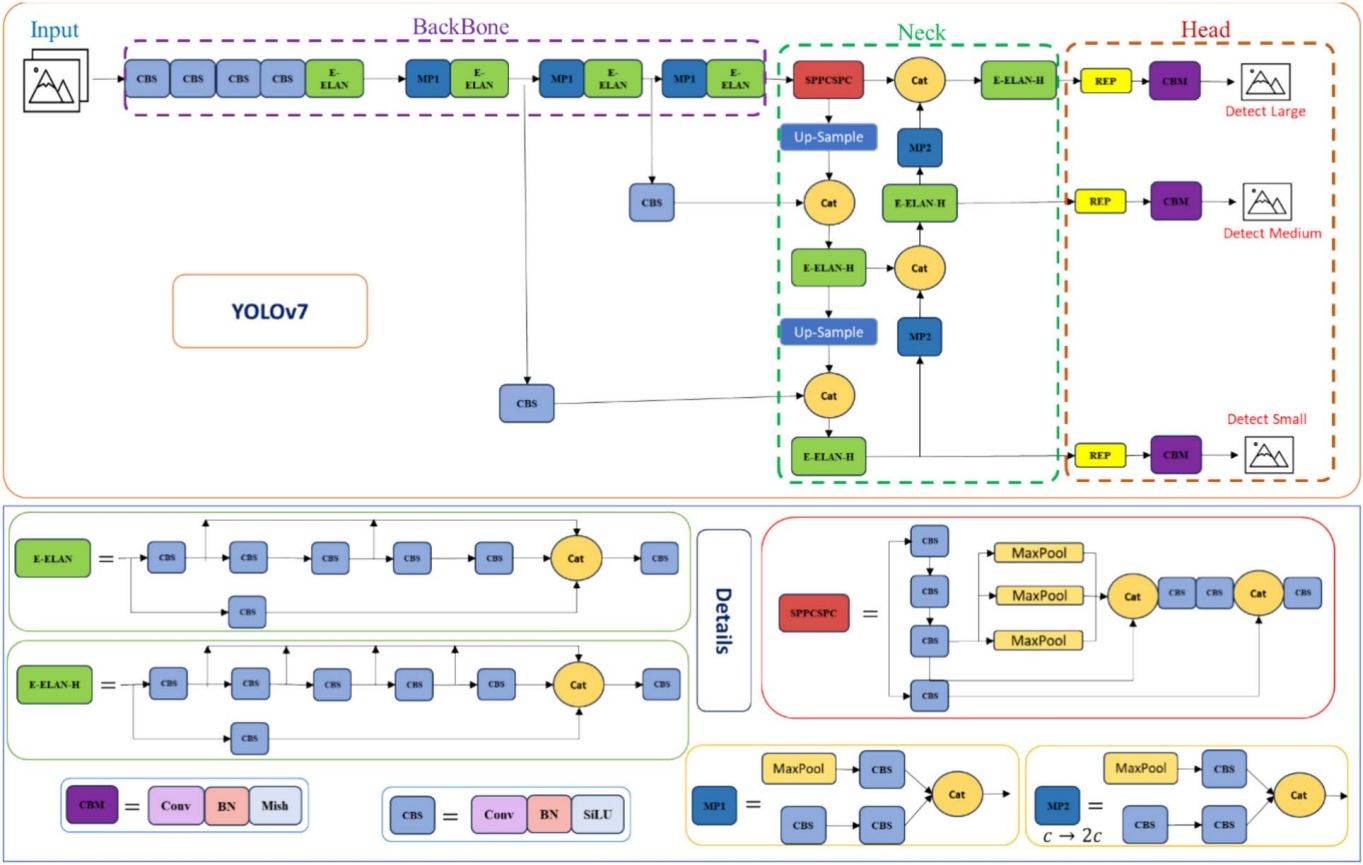
The YOLOv7 network structure.

YOLOv7 is a huge step forward in the field of object identification, recognized as a milestone for its extraordinary speed and accuracy. This sophisticated model is part of the YOLO (You Only Look Once) family, which is notable for its single-stage detection approach that detects both the positions and classes of objects while generating boundaries surrounding them in real time. The structure of YOLOv7 reflects the YOLO family’s continual growth. It builds on the foundations provided by YOLOv5, YOLOv4, YOLO-R, and Scaled YOLOv4, including the greatest aspects of each while making major enhancements^56^. The core of YOLOv7 is a CNN that creates picture features, commonly known as embeddings. The method uses a single CNN to process the full picture, partition it into grids, and provide predictions. YOLO has three main components: the backbone, neck, and head. The backbone extracts attributes from input photos, the neck creates pyramids of features, and the head detects the output. The creators of YOLOv7 made significant architectural improvements, including E-ELAN (Extended Efficient Layer Aggregation Network), model scaling, planned reparametrized, coarseness for auxiliary loss, and fineness for lead loss^57^. The E-ELAN is a fundamental architectural component of YOLOv7 that extends the notion of ELAN to optimize the network’s learning capabilities while preserving the original gradient route. This optimization is further increased by employing the technique of compound model scaling, which adapts the dimension, resolution, and width of the model to meet the varied demands of the application and decreases the computational complexity^57^. Reparameterization is an after-training strategy for models that extends learning and enhances prediction. There are two sorts of combinations: model-level and module-level. RepConv, similar to Resnet, uses two identity connections with a 1 × 1 filter in between. YOLOv7 uses RepConvN without an identity connection in its reparametrized architecture to eliminate duplication when employing convolutional layers that work with concatenation or residuals. In YOLOv7, several heads play unique roles: the lead head provides the final output, while the auxiliary head supports training. A two-label assigner procedure combines lead head-guided and coarse-to-fine approaches^58^. Soft labels, which are created from lead predictions and ground truth, dynamically modify data granularity throughout training. This method improves learning capacity by capturing the dispersion of data and correlation efficiently. Figure 7 represents the network structure of Yolov7.

#### YOLOv8

**Fig. 8:**
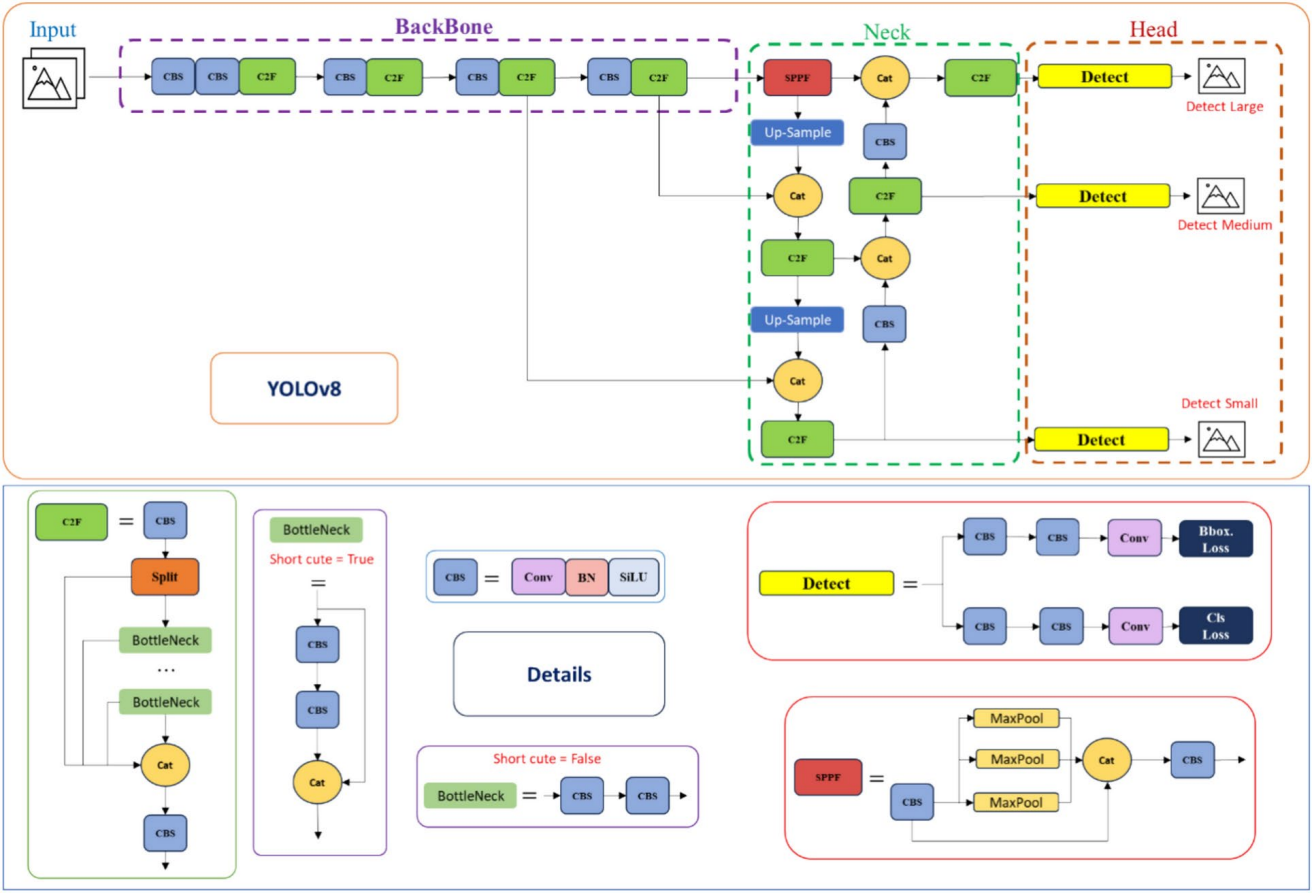
The YOLOv8 network structure.

YOLOv8 is one of the most recent updates to the YOLO (You Only Look Once) line of object-detection systems, which are noted for their real-time speed and superior precision in computer vision applications. Developed by Ultralytics, YOLOv8 is intended to process photos in just one step, which benefits its effectiveness and speed, making it a great option for real-time applications. Ultralytics improved YOLOv8, which made it easier to utilize than YOLOv5^59^. This enhanced model enhances YOLOv5’s capability and user-friendliness for computer vision jobs. YOLOv8 brings numerous significant improvements over its predecessors. It can recognize objects, segment instances, and classify images, increasing its application area. The model employs anchor boxes to increase the accuracy of bounding box predictions, and it may be fine-tuned on customized datasets to enhance its performance for particular applications^60^. The building’s structure is divided into two main parts: the backbone and the head. The backbone is built on an updated CSPDarknet53 with cross-stage partial connections, and it contains a novel C2f. module for improved extraction of features and an SPPF module for effective spatial feature pooling. The head evaluates maps of features to anticipate bounding boxes and object classes. YOLOv8 abandons anchor-based detection in favor of an anchor-free method, which simplifies the model and accelerates Non-Maximum Suppression (NMS) post-processing. The C2f. combines the outcomes of all bottleneck levels to provide a richer representation of features, in contrast to YOLOv5’s C3 layer, which only employs the result of the final bottleneck layer^60,61^. This adjustment, together with an increase in the size of the kernel of the initial convolution stage in the bottleneck, enables enhanced feature integration without extending the parameter number resulting in a better-performing and more precise object identification model^60,61^. The YOLOv8 model is both accurate and efficient, with a lightweight design that prioritizes speed. It has demonstrated considerable performance increases on the COCO dataset, outperforming competing models in terms of accuracy and speed. The model’s performance is further improved by the small number of parameters and FLOPs. Figure 8 represents the network structure of Yolov8.

### The combination of the optimization algorithms and YOLOs

To improve object detection performance, the article explores the complex process of combining different metaheuristic optimization techniques with the YOLOv7 and YOLOv8 models in this section. The YOLO architecture in conjunction with these optimization methods is a key factor in determining the effectiveness and accuracy of object recognition in remote-sensing photos. The utilized approaches to combine the metaheuristic optimization techniques with YOLOv7 and YOLOv8 can be described as:

1. **Initialization and configuration of parameters:** The hyperparameters of the YOLO models were initialized and configured using each metaheuristic algorithm. This involves adjusting crucial parameters that affect how neural networks are trained.
2. **Iterative parameter tuning:** Throughout the training stage, the optimization algorithms made iterative adjustments to the hyperparameters. The YOLO hyperparameters were dynamically modified by the application of optimization methods. The models were able to converge to optimal solutions more quickly according to this iterative tuning procedure, which improved the accuracy of object detection.
3. **Evaluation of the fitness function:** The article employed a fitness function based on mAP, recall, and precision to evaluate the YOLO models’ performance. The objective for each metaheuristic algorithm was to minimize this fitness function. The algorithms successfully directed the YOLO models toward configurations that maximized detection performance by continuously evaluating the fitness function.
4. **Algorithm-specific enhancements**: Each metaheuristic algorithm brought unique enhancements to the YOLO models. Overall detection efficiency and precision increased as a result of these algorithm-specific improvements.

Through the previously indicated approaches, the study was able to successfully integrate these metaheuristic optimization techniques with the YOLOv7 and YOLOv8 models, improving the overall performance, recall rates, and precision of object detection in remote sensing photos. These contributions highlight how well the proposed method works for tackling the difficult problems associated with object detection in the context of remote sensing imaging.

## Experimental results

### Computational environments

The experiments are performed through Google Collab pro-environment, with a V100 GPU processor equipped with 16 GB of GPU RAM, 51 GB of system RAM, and 201 GB disk space and programmed using the Python programming language.

### Performance metrics

The evaluation of operational efficiency in detecting objects from remote sensing photos includes numerous critical measures, such as recall, precision, and Mean Average accuracy (mAP). These metrics jointly assess the model’s ability to detect objects, providing precise information about its effectiveness. True positives (TP) are objects accurately recognized as members of the target class, whereas true negatives (TN) are objects correctly identified as non-members. False positives (FP) are objects that are incorrectly identified as belonging to the target class, whereas false negatives (FN) are objects that belong to the target class but are incorrectly classified elsewhere. Consequently, the following evaluation metrics were computed^62–64^:

1. **Recall (R)** is measured as the ratio of true positively classified pixels to all positively classified pixels.
  41 $$R= \frac{TP}{FN+TP}$$
2. **Precision (P)** is measured as the number of true positive pixel classifications divided by the total number of the algorithm’s positive pixel classifications.
  42 $$P= \frac{TP}{TP+FP}$$
3. **Average precision (AP)** is equivalent to the area under the precision-recall curve.
  43 $$AP= {\int }_{0}^{1}P\left(R\right) dR$$
4. The AP value across several categories is represented by **Mean Average Precision (mAP),** where 'c' indicates the number of categories.
  44 $$mAP=\frac{1}{c}{\sum }_{j=1}^{c}{AP}_{j}$$

Equation (44) uses the form ${AP}_{j}$ to represent the AP with class index number j, and c to represent the number of sample classes in the dataset (4 and 10 in this article). When the Intersection over Union (IoU) of the model used for detection has been adjusted to 0.5, the mean precision is indicated by mAP 0.5, whereas when the IoU of the detecting model is set between 0.5 and 0.95 (with measurements obtained at intervals of 0.5), the average precision is shown by mAP 0.5:0.95.

5- ** Fitness Score** is the output value of the fitness function that indicates the performance of the model during the testing phase which gives an indication of the hyperparameters to be selected during the optimization of hyperparameters stage.
  45 $$Fit Score=1-(\boldsymbol{ }0.9 \times \left(\text{mAP }0.5:0.95\right)+0.1 \times \left(\text{mAP }0.5\right))$$

### The loss function

#### YOLOv7

In YOLOv7, the loss function typically consists of several components designed to optimize object detection performance. The primary components usually include^56–58^:

- **Coordinates loss**: Measures how accurately the model predicts the bounding box coordinates of objects.
- **Confidence loss**: Evaluate the confidence score for object presence in the bounding box and how well it matches the ground truth.
- **Class loss**: Computes the classification loss for predicting the class labels of detected objects.

YOLOv7 Equation can be written as:

46 $$\begin{aligned} & Loss = \lambda_{coord} \mathop \sum \limits_{i = 0}^{{S^{2} }} \mathop \sum \limits_{j = 0}^{B} 1_{ij}^{obj} \left[ {\left( {x_{i} - \hat{x}_{i} } \right)^{2} + \left( {y_{i} - \hat{y}_{i} } \right)^{2} } \right] \\ & \quad + \lambda_{coord} \mathop \sum \limits_{i = 0}^{{S^{2} }} \mathop \sum \limits_{j = 0}^{B} 1_{ij}^{obj} \left[ {\left( {\sqrt {w_{i} } - \sqrt {\hat{w}_{i} } } \right)^{2} + \left( {\sqrt {h_{i} } - \sqrt {\hat{h}_{i} } } \right)^{2} } \right] \\ & \quad + \lambda_{obj} \mathop \sum \limits_{i = 0}^{{S^{2} }} \mathop \sum \limits_{j = 0}^{B} 1_{ij}^{obj} \left( {C_{i} - \hat{C}_{i} } \right)^{2} + \lambda_{noobj} \mathop \sum \limits_{i = 0}^{{S^{2} }} \mathop \sum \limits_{j = 0}^{B} 1_{ij}^{obj} \left( {C_{i} - \hat{C}_{i} } \right)^{2} \\ & \quad + \lambda_{cls} \mathop \sum \limits_{i = 0}^{{S^{2} }} 1_{ij}^{obj} \mathop \sum \limits_{c \in classes} \left( {p_{i} \left( c \right) - \hat{p}_{i} \left( c \right)} \right)^{2} \\ \end{aligned}$$

where, ${S}^{2}$ represents the number of grid sizes, $B$ represents the number of bounding boxes predicted per grid cell, (${x}_{i}$, ${y}_{i}$*,* ${w}_{i}$, ${h}_{i}$) represent the predicted coordinates of boundary box x and y axis of the center localization, box width, and box height respectively, (${\widehat{x}}_{i}, {\widehat{y}}_{i}, {\widehat{w}}_{i}, {\widehat{h}}_{i}$) represent the ground truth coordinates of boundary box x and y axis of the center localization, box width, and box height respectively, ${C}_{i}$ is the predicted score of confidence, ${\widehat{C}}_{i}$ is the ground truth score of confidence, ${p}_{i}(c)$ represents the predicted probability, ${\widehat{p}}_{i}(c)$ is the ground truth probability, and (${\lambda }_{coord}$, ${\lambda }_{obj}$, ${\lambda }_{noobj}$, ${\lambda }_{cls}$) are coefficients that balance the contribution of each loss component confidence to the overall loss.

#### YOLOv8

In Yolov8, the loss function is an important aspect of training the model for reliable object detection. It has three major components: bounding box loss, classification loss, and objectness loss. The bounding box loss is a hybrid of Distribution Focal Loss (DFL) and Complete Intersection over Union (CIoU) losses^59,60^. DFL strengthens the model’s capacity to predict correct bounding boxes for objects with ambiguous boundaries, whereas CIoU loss improves overall bounding box prediction accuracy^60,61^. YOLOv8 employs Cross Entropy loss for classification, which evaluates the error made in predicting the class to which each observed object falls. Previous versions of YOLO included a distinct objectness loss, but in YOLOv8, the objectness confidence is integrated into the IoU-Aware Classification Score, eliminating the requirement for a separate loss component. The final loss is the weighted total of the three separate losses. The weights determine the relative value or contribution of each loss to the ultimate total loss, which is optimized during training. YOLOv8 Equation can be written as:

47 $$loss= \alpha {loss}_{box}+\beta {loss}_{dfl}+\gamma {loss}_{cls}$$

where, α, β, and γ represent the corresponding loss weight proportion, ${loss}_{box}$ is boundary box loss, ${loss}_{dfl}$ is distribution focal loss, and ${loss}_{cls}$ represents the classification loss.

### Optimized hyperparameters

During the experiments the study chooses various parameters to be optimized for YOLOv7 and YOLOv8 to reach the best performance these parameters are described and represented in Table 4, while the study chooses to leave some other parameters constant as batch size equal to 16, with a number of epochs equal to 25, and input image size equal to 640 × 640. Furthermore, all the optimization algorithm populations are equal to 3 and the number of iterations is equal to 20 due to the high computational cost and the huge running time.

**Table 4:** The ranges of the optimized parameters.

Parameter	Model		Range (Step)	Parameter description
	v7	v8		
**Lr0**	**√**	**√**	**0.001–0.1** (0.0025)	The starting rate of learning, modifying this number is essential to the optimization process since it affects the rate at which model weights are changed
**Lrf**	**√**	**√**	**0.001–0.1** (0.0025)	The final Learning rate
**Momentum**	**√**	**√**	**0.5–1** (0.05)	Momentum factor affecting the current update’s incorporating previous gradients
**Weight_Decay**	**√**	**√**	**0.00025–0.001** (0.0025)	To prevent overfitting, the L2 regularization term penalizes large weights
**Warmup_Epochs**	**√**	**√**	**1–5** (1)	The number of learning rate warm-up epochs is used to progressively raise the learning rate from a low value to the starting learning rate to stabilize training at an early stage
**Warmup_Momentum**	**√**	**√**	**0.2–1** (0.1)	starting momentum during the warm-up phase and progressively bringing it up towards the predetermined momentum during the warm-up
**Warmup_Bias_Lr**	**√**	**√**	**0–0.3** (0.05)	Throughout the warm-up stage, the learning rate for bias characteristics assists in stabilizing model training in the early epochs
**Box**	**√**	**√**	**6–9** (1)	The weighting of the box’s loss factor in the loss function determines the degree to which precise prediction of bounding box coordinates is valued
**Cls**	**√**	**√**	**0.1–1.1** (0.1)	The relevance of accurately predicting the class about other components is determined by the weight of the classification loss in the overall loss function
**Cls_Pw**	**√**		**0.5–2** (0.25)	this hyperparameter stands for the binary cross-entropy (BCE) loss function’s positive weight. It establishes the weighted relevance of positive training samples
**Obj**	**√**		**0–2** (0.25)	The gain for the object loss is represented by this hyperparameter. It adjusts the object loss according to the pixel size of the item
**Obj_Pw**	**√**		**0.5–2** (0.25)	The positive weight for the BCE loss function in the abjectness prediction is represented by this hyperparameter. It establishes the weighted relevance of positive training samples
**Dfl**		**√**	**0.5–3** (0.25)	Weight of the distribution of focal loss, which is utilized for fine-grained categorization in some YOLO versions
**Iou_T**	**√**		**0.1–0.7** (0.1)	The training threshold for intersection over union, or IoU, is represented by this hyperparameter. It is employed to ascertain if a projected bounding box is regarded as a positive or negative prediction. Positive predictions are those that have a predicted bounding box with an IoU value higher than the threshold
**Anchor_T**	**√**		**3–8** (1)	This hyperparameter stands for the threshold of anchor multiple. During training, it is utilized to filter out anchor boxes with little overlap with ground truth boxes
**Anchors**	**√**		**3–10** (1)	The number of anchors per output layer is indicated by this hyperparameter. It establishes how many anchor boxes are utilized in object detection
**Fl_Gamma**	**√**		**0–2** (0.25)	The focal loss function’s gamma value is represented by this hyperparameter. It modifies the weight assigned to challenging cases during training

The range of values are in bold.

### Computational configures for training

This study evaluated the performance improvements of YOLOv7 and YOLOv8 models using several metaheuristic optimization strategies. The main measures of interest were recall, precision, and mean Average Precision (mAP), which directly represent the improvements’ effectiveness in improving object detection performance. The optimization techniques used in the studies (GA, AO, ROA, PSO, GWO, and HPSGWO) do not change the design of the YOLO models. Instead, they modify the hyperparameters and weights of the preset structure. As a result, no matter what optimization is used, the number of parameters and GFLOPs (Giga Floating Point Operations per second) stay constant for each YOLO version. As a result, while computational measures like GFLOPs and GPU utilization are important, they do not differ significantly among different YOLO version optimizations. To provide a comprehensive view of the computational aspects, this section presents the number of parameters, GFLOPs, and GPU utilization for the proposed YOLOv7 and YOLOv8 models in Table 5.

**Table 5:** The training configurations of YOLOv7 and YOLOv8.

Model	Number of parameters (M)	GFLOPs	GPU Utilized (GB)
YOLOv7	37.2	105.4	15.9
YOLOv8	11.4	29.7	4.2

### Experiments

In the experimental section, a comprehensive comparative analysis was conducted between YOLOv7 and YOLOv8 in conjunction with six metaheuristic optimization algorithms GWO, PSO, GA, ROA, AO, and HPSGWO. This comparison was executed across two distinct remote sensing datasets, namely RSOD and VHR-10, to elucidate the efficacy of these optimization techniques in enhancing the performance of various YOLO algorithms.

In all the experiments while the study chooses various parameters to be optimized mentioned in section "The loss function", the study leaves some other parameters constant as batch size equal to 16, number of epochs equal to 25, and input image size equal to 640 × 640. Furthermore, all the optimization algorithm populations are equal to 3 and the number of iterations is equal to 20 due to the high computational cost and the huge running time. Train, validation, and test percentage are 70%, 15%, and 15%. These values were selected based on numerous preliminary experiments to ensure optimal performance and efficiency.

### RSOD dataset

**Fig. 9:**
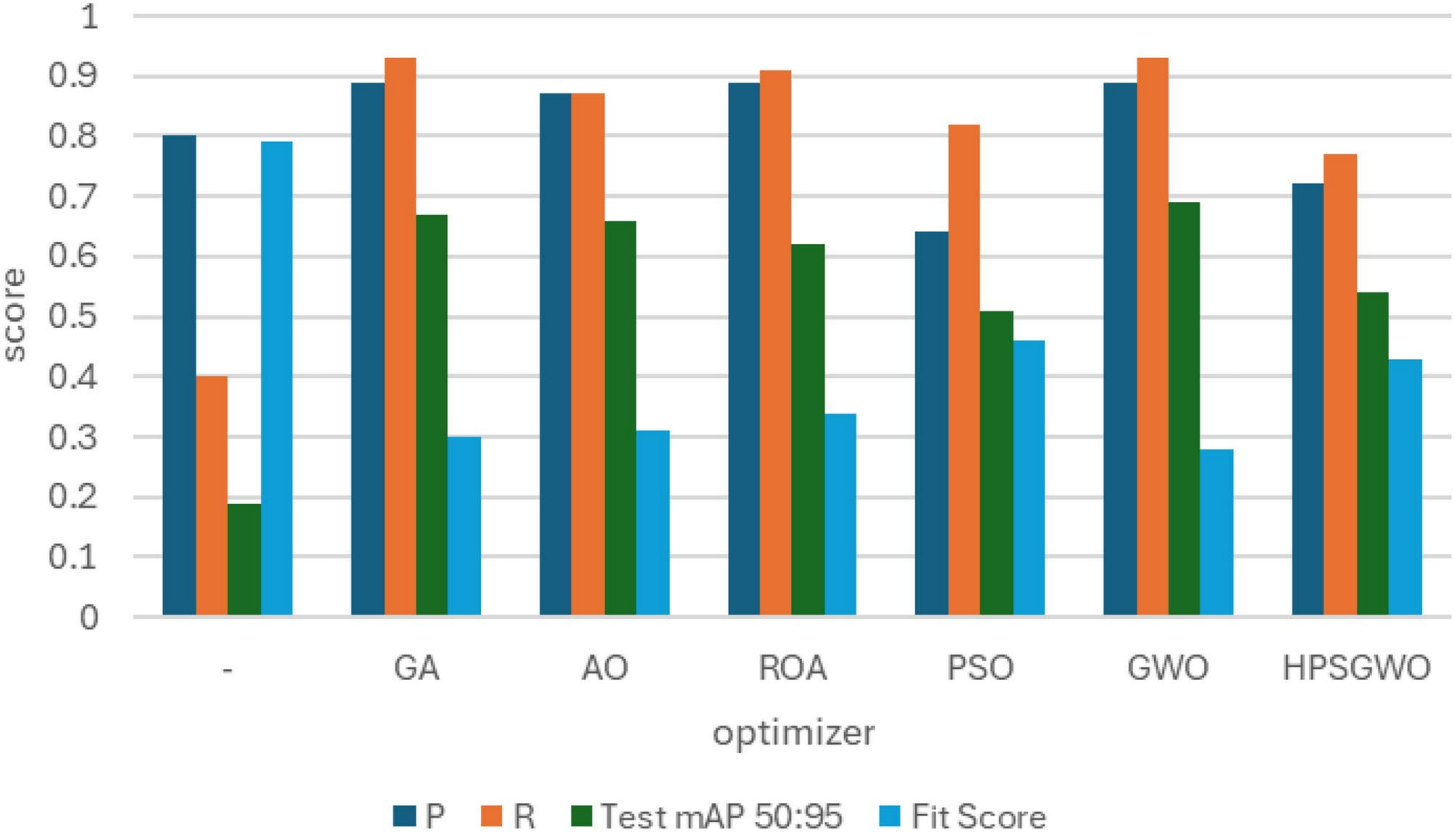
visual comparison of optimizers’ performance with YOLOv7 on the RSOD dataset.

**Fig. 10:**
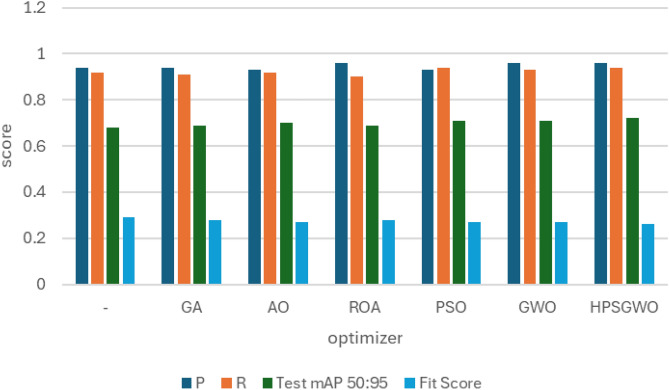
Visual comparison of optimizers’ performance with YOLOv8 on the RSOD dataset.

In the first experiment, YOLOv7 and YOLOv8 performance have been enhanced by six distinct metaheuristic optimization strategies and examined on the RSOD dataset. The performance of the proposed optimization algorithms has been compared in terms of recall, precision, and mAP. This study’s main objective is to investigate the effectiveness of various optimization strategies on the YOLO algorithms’ accuracy and performance when applied to the RSOD dataset. The 14 different combination output performance metrics on the RSOD dataset are represented in Table 6. Furthermore, a visual comparison of the optimizer’s performance with YOLOv7 is represented in Fig. 9, while a visual comparison of the optimizer’s performance with YOLOv8 is represented in Fig. 10.

**Table 6:** The performance metrics of the different optimized YOLO versions on the RSOD dataset.

Optimizer	P	R	Train mAP 50	Train mAP 50:95	Val mAP 50	Val mAP 50:95	Test mAP 50	Test mAP 50:95	Fit Score
**YOLOv7**									
**–**	0.8	0.4	0.4	0.21	0.38	0.2	0.38	0.19	0.79
**GA**	0.89	0.93	0.97	0.68	0.95	0.66	0.95	0.67	0.3
**AO**	0.87	0.87	0.95	0.68	0.94	0.66	0.95	0.66	0.31
**ROA**	0.89	0.91	0.93	0.64	0.94	0.64	0.93	0.62	0.34
**PSO**	0.64	0.82	0.85	0.57	0.83	0.5	0.82	0.51	0.46
GWO	**0.89**	**0.93**	**0.97**	**0.69**	**0.96**	**0.69**	**0.96**	**0.69**	**0.28**
**HPSGWO**	0.72	0.77	0.89	0.60	0.83	0.54	0.82	0.54	0.43
**YOLOv8**									
**–**	0.94	0.92	0.96	0.69	0.96	0.69	0.96	0.68	0.29
**GA**	0.94	0.91	0.96	0.69	0.95	0.68	0.96	0.69	0.28
**AO**	0.93	0.92	0.95	0.7	0.96	0.69	0.95	0.7	0.27
**ROA**	0.96	0.90	0.96	0.68	0.96	0.69	0.96	0.69	0.28
**PSO**	0.93	0.94	0.97	0.72	0.97	0.7	0.96	0.71	0.27
**GWO**	0.96	0.93	0.97	0.72	0.96	0.71	0.96	0.71	0.27
HPSGWO	**0.96**	**0.94**	**0.97**	0.71	**0.97**	**0.72**	**0.97**	**0.72**	**0.26**

Best values are in bold.

**Fig. 11:**
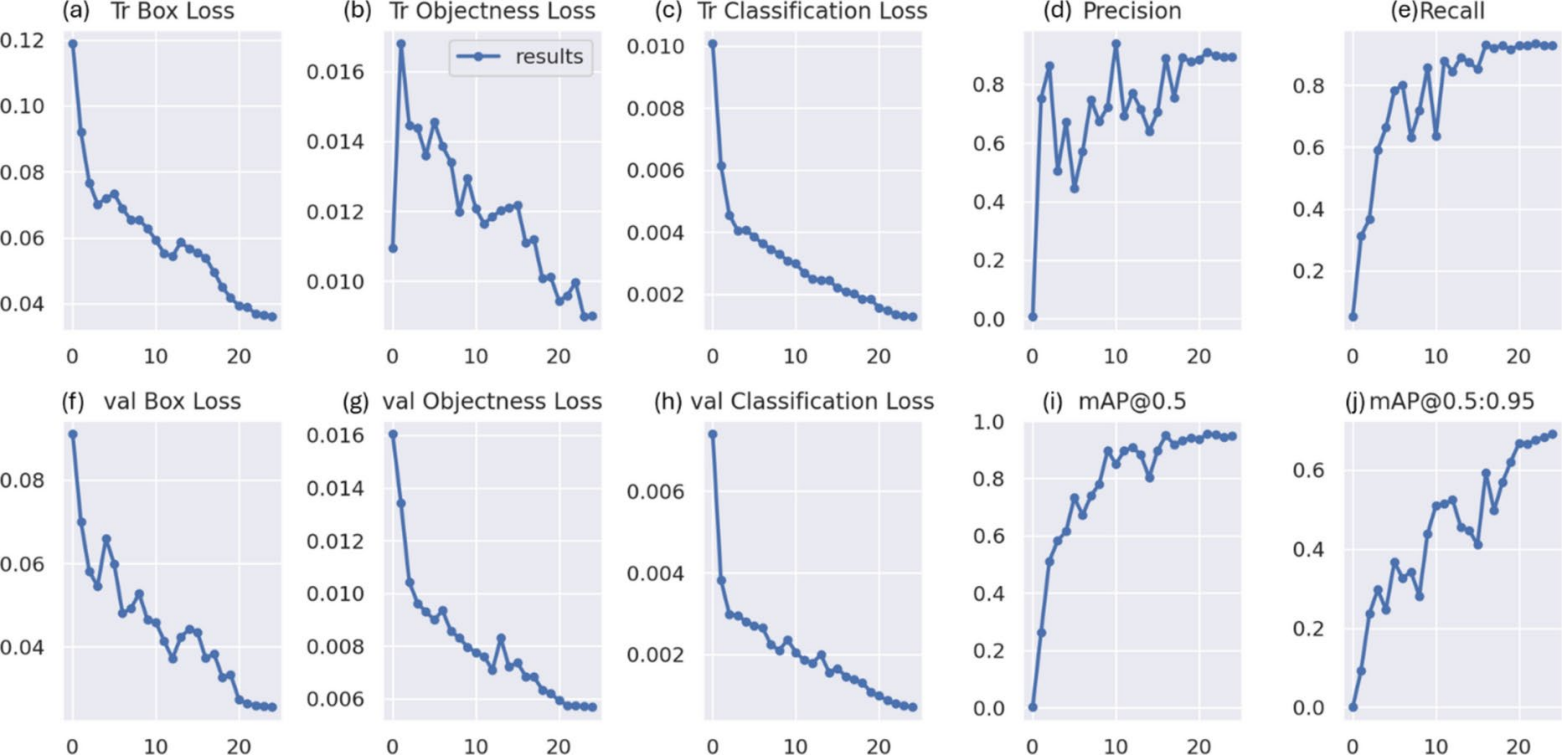
the YOLOv7 optimized by GWO performance metrics on RSOD dataset; (**a**) training box loss, (**b**) training objectness loss, (**c**) training classification loss, (**d**) precision curve, (**e**) recall curve, (**f**) validation box loss, (**g**) validation objectness loss, (**h**) validation classification loss, (**i**) training mAP 0.5, (**j**) training mAP 0.5:0.95.

**Fig. 12:**
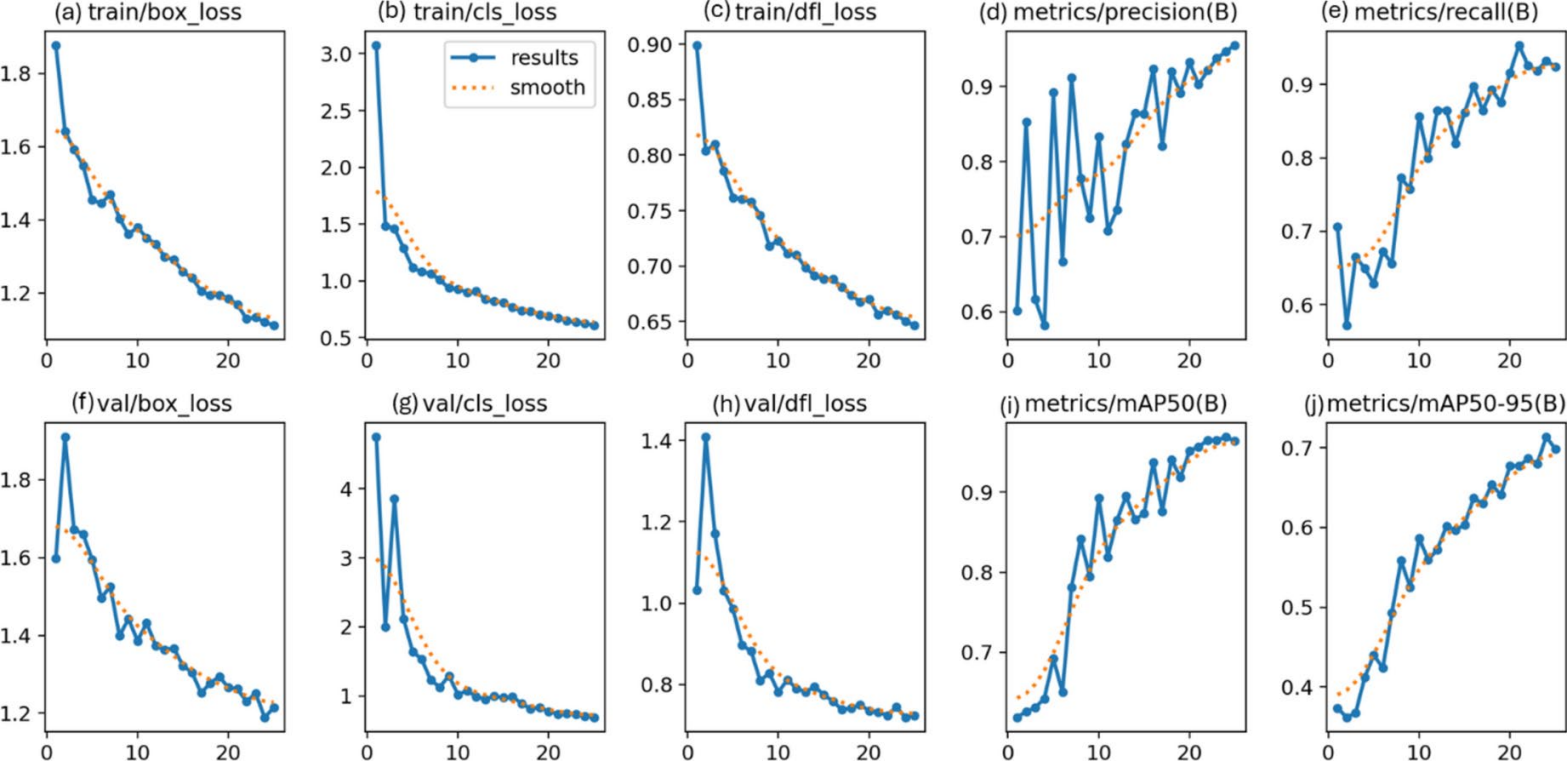
the YOLOv8 optimized by HPSGWO performance metrics on RSOD dataset; (**a**) training box loss, (**b**) training cls loss, (**c**) training dfl loss, (**d**) precision curve, (**e**) recall curve, (**f**) validation box loss, (**g**) validation cls loss, (**h**) validation dfl loss, (**i**) training mAP 0.5, (**j**) training mAP 0.5:0.95.

Based on six alternative metaheuristic algorithms (GA, AO, ROA, PSO, GWO, and HPSGWO), the experimental findings offer a thorough comparison of the performance of YOLOv7 and YOLOv8 on the RSOD dataset. The findings in Table 6 show that using metaheuristic optimization techniques improves YOLOv7 and YOLOv8 performance considerably. After using the GWO method to optimize YOLOv7, the precision increased from 0.8 to 0.89, and the recall from 0.4 to 0.93. In a similar vein, YOLOv8’s precision increased from 0.94 to 0.96 and its recall from 0.92 to 0.93 after optimization using the HPSGWO technique. Based on the fit score, the best model for YOLOv7 is the one optimized with the GWO optimization algorithm. This optimized model achieved a fit score of 0.28, indicating its effectiveness in enhancing the accuracy and performance of YOLOv7 on the RSOD dataset. On the other hand, for YOLOv8, the best model based on the fit score is the one optimized with the HPSGWO algorithm. This optimized model achieved a fit score of 0.26, showcasing its ability to improve the accuracy and performance of YOLOv8 on the RSOD dataset. Furthermore, it’s interesting to note that in certain instances, the non-optimized YOLOv8 model scored worse than the optimized YOLOv7 model. As an illustration, the fit score of 0.28 for the GWO-optimized YOLOv7 model is lower than the 0.29 value for the non-optimized YOLOv8 model. This implies that YOLOv7’s performance may be much improved by the optimization procedures, enabling it to compete with YOLOv8. But when comparing the two models’ optimized versions, YOLOv8 consistently performs better than YOLOv7 in every performance indicator. This is consistent with the way the YOLO family has evolved, with each new version improving upon the last to fix bugs and boost functionality. Comprehensive graphics showing precision-recall curves and other metrics like losses and mAPs are included in addition to the performance measures previously addressed, providing a more thorough assessment of the optimized YOLOv7 and YOLOv8 models. These additional investigations shed a greater spotlight on how metaheuristic optimization techniques affect the precision and effectiveness of the object detection systems used with the RSOD dataset. Figure 11 represents the YOLOv7 with GWO optimizer performance metrics (best optimized YOLOv7), and Fig. 12 the YOLOv8 with HPSGWO optimizer performance metrics (best optimized YOLOv8).

**Fig. 13:**
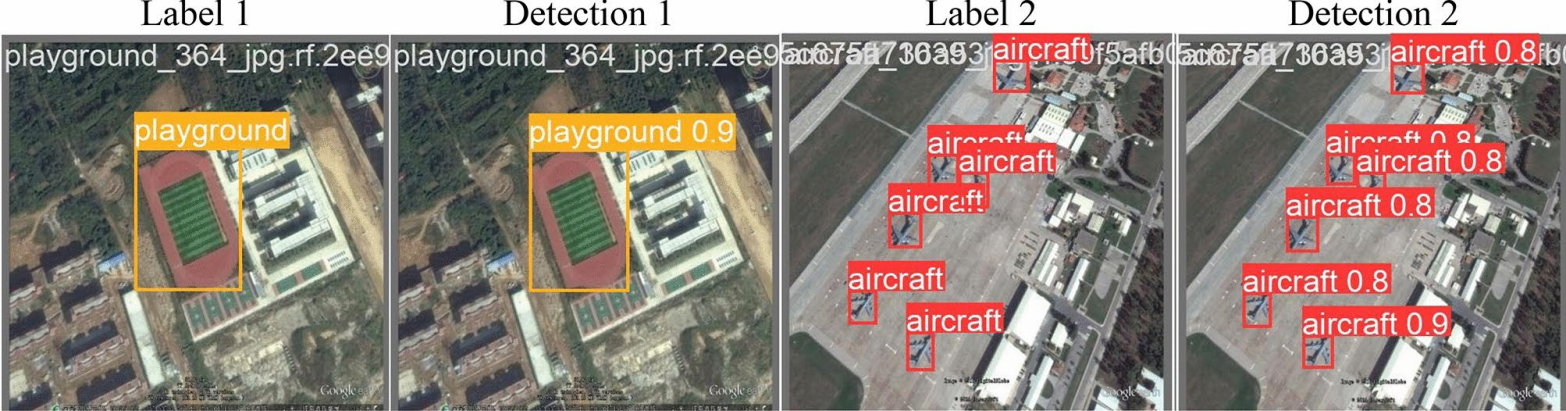
The detection results of the RSOD dataset test samples using YOLOv8 optimized by HPSGWO.

To sum up, the experimental findings show that metaheuristic optimization methods are useful for improving YOLO algorithms’ performance. GWO seems to be the best optimization method out there for YOLOv7 while having a great result with YOLOv8, and HPSGWO is the best optimization method for YOLOv8. These results offer important new information for the creation of object detection systems that are more precise and effective. Figure 13 represents the detection results of the RSOD dataset test samples using YOLOv8 optimized by HPSGWO.

### VHR-10 dataset

**Fig. 14:**
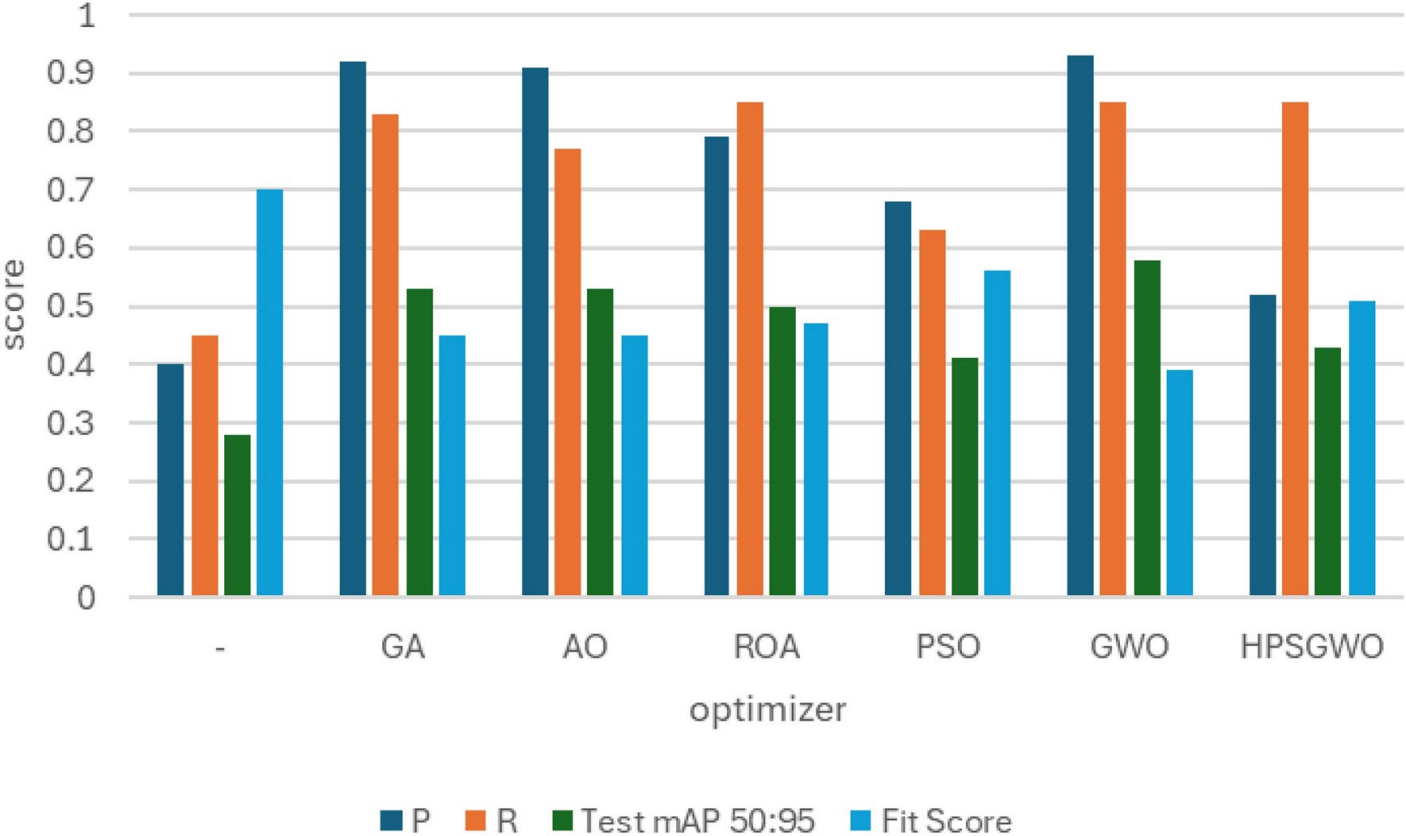
Visual comparison of optimizers performance with YOLOv7 on the VHR-10 dataset.

**Fig. 15:**
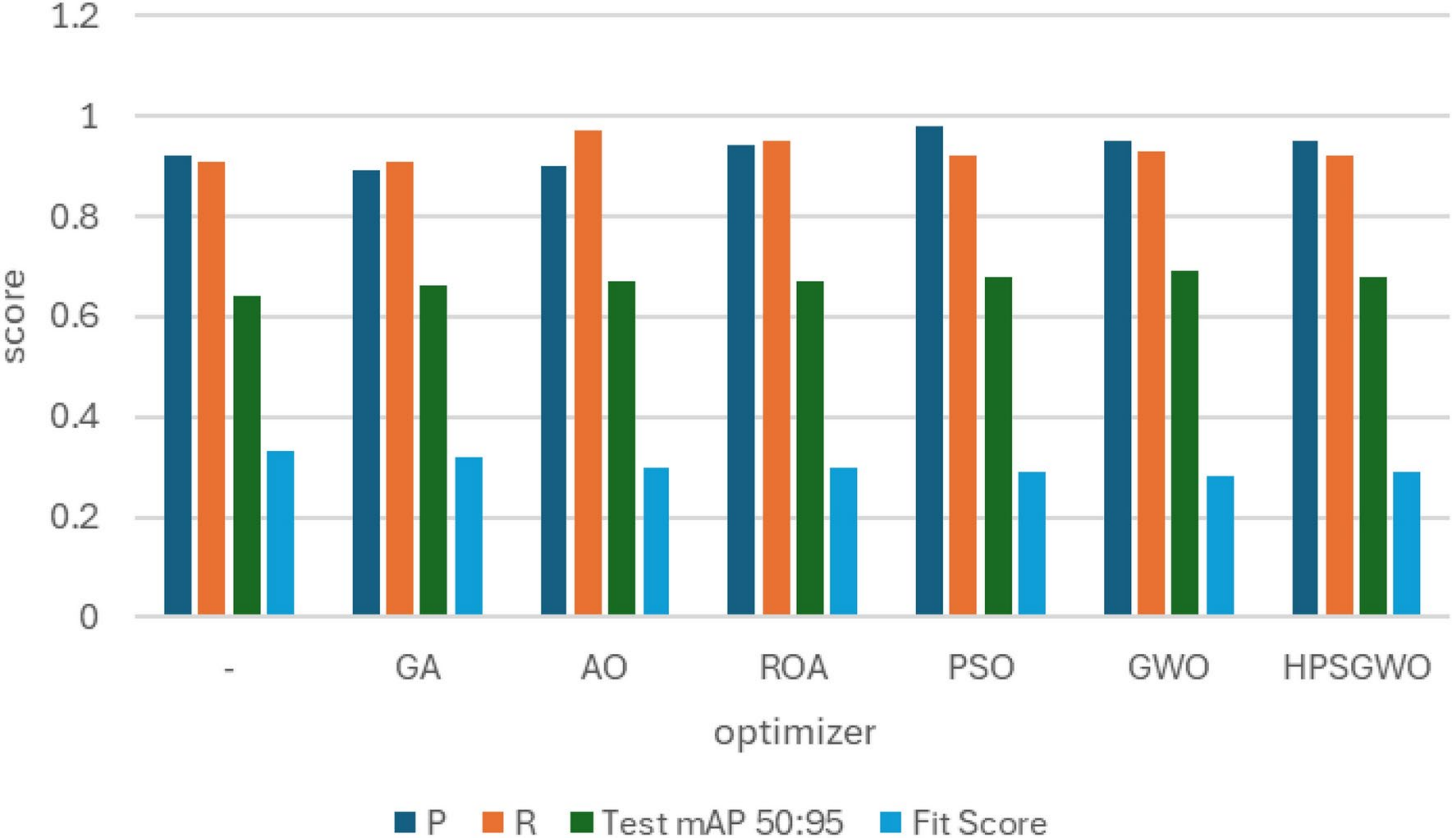
Visual comparison of optimizers performance with YOLOv8 on the VHR-10 dataset.

Subsequently, to ensure the findings of the RSOD dataset experiment outcomes the experimental attention is shifted to the VHR-10 dataset, and a detailed study is carried out to evaluate the effectiveness of six alternative metaheuristic optimization methods combined with YOLOv7 and YOLOv8. This study’s objective is to assess how different optimization strategies affect the YOLO algorithms’ accuracy and efficiency when used with the VHR-10 dataset which has 10 different classes. The 14 different combination outputs on the VHR-10 dataset are represented in Table 7. Furthermore, a visual comparison of the optimizer’s performance with YOLOv7 is represented in Fig. 14, while a visual comparison of the optimizer’s performance with YOLOv8 is represented in Fig. 15.

**Table 7:** The performance metrics of the different optimized YOLO versions on the VHR-10 dataset.

Optimizer	P	R	Train mAP 50	Train mAP 50:95	Val mAP 50	Val mAP 50:95	Test mAP 50	Test mAP 50:95	Fit Score
**YOLOv7**									
**–**	0.4	0.45	0.41	0.29	0.42	0.29	0.41	0.28	0.7
**GA**	0.92	0.83	0.87	0.56	0.78	0.55	0.77	0.53	0.45
**AO**	0.91	0.77	0.82	0.55	0.79	0.54	0.76	0.53	0.45
**ROA**	0.79	0.85	0.86	0.52	0.85	0.49	0.85	0.5	0.47
**PSO**	0.68	0.63	0.74	0.46	0.71	0.43	0.72	0.41	0.56
GWO	**0.93**	**0.85**	**0.87**	**0.58**	**0.87**	**0.56**	**0.87**	**0.58**	**0.39**
**HPSGWO**	0.52	0.85	0.77	0.44	0.75	0.44	0.72	0.43	0.51
**YOLOv8**									
**–**	0.92	0.91	0.97	0.65	0.96	0.65	0.96	0.64	0.33
**GA**	0.89	0.91	0.97	0.65	0.97	0.63	0.95	0.66	0.32
**AO**	0.90	0.97	0.98	0.66	0.99	0.67	0.99	0.67	0.3
**ROA**	0.94	0.95	0.98	0.67	0.97	0.66	0.98	0.67	0.3
**PSO**	0.98	0.92	0.99	0.69	0.99	0.68	0.99	0.68	0.29
GWO	**0.95**	**0.93**	**0.99**	**0.70**	**0.98**	**0.68**	**0.99**	**0.69**	**0.28**
**HPSGWO**	0.95	0.92	0.97	0.68	0.96	0.68	0.97	0.68	0.29

Best values are in bold.

**Fig. 16:**
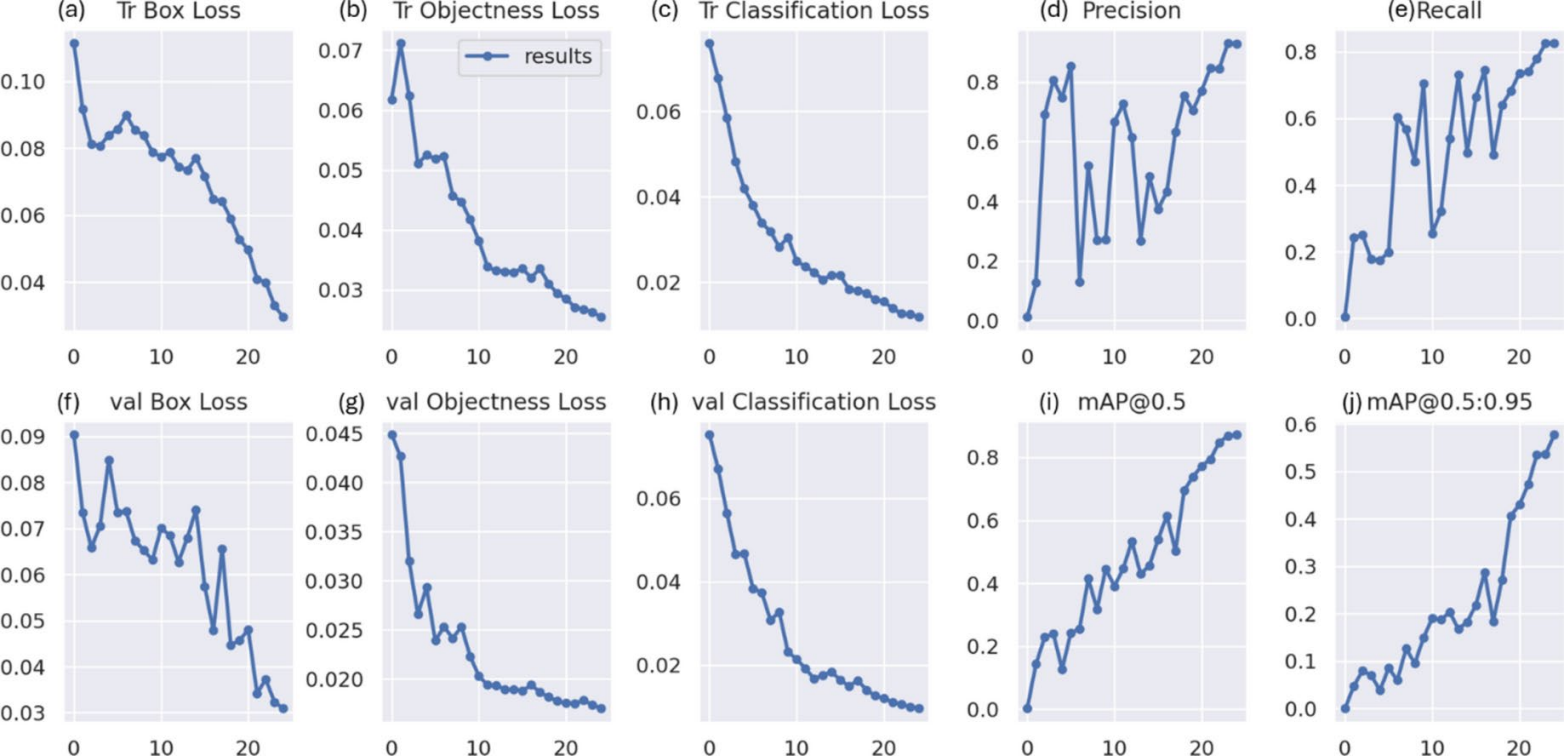
The YOLOv7 optimized by GWO performance metrics on VHR-10 dataset; (**a**) training box loss, (**b**) training objectness loss, (**c**) training classification loss, (**d**) precision curve, (**e**) recall curve, (**f**) validation box loss, (**g**) validation objectness loss, (**h**) validation classification loss, (**i**) training mAP 0.5, (**j**) training mAP 0.5:0.95.

**Fig. 17:**
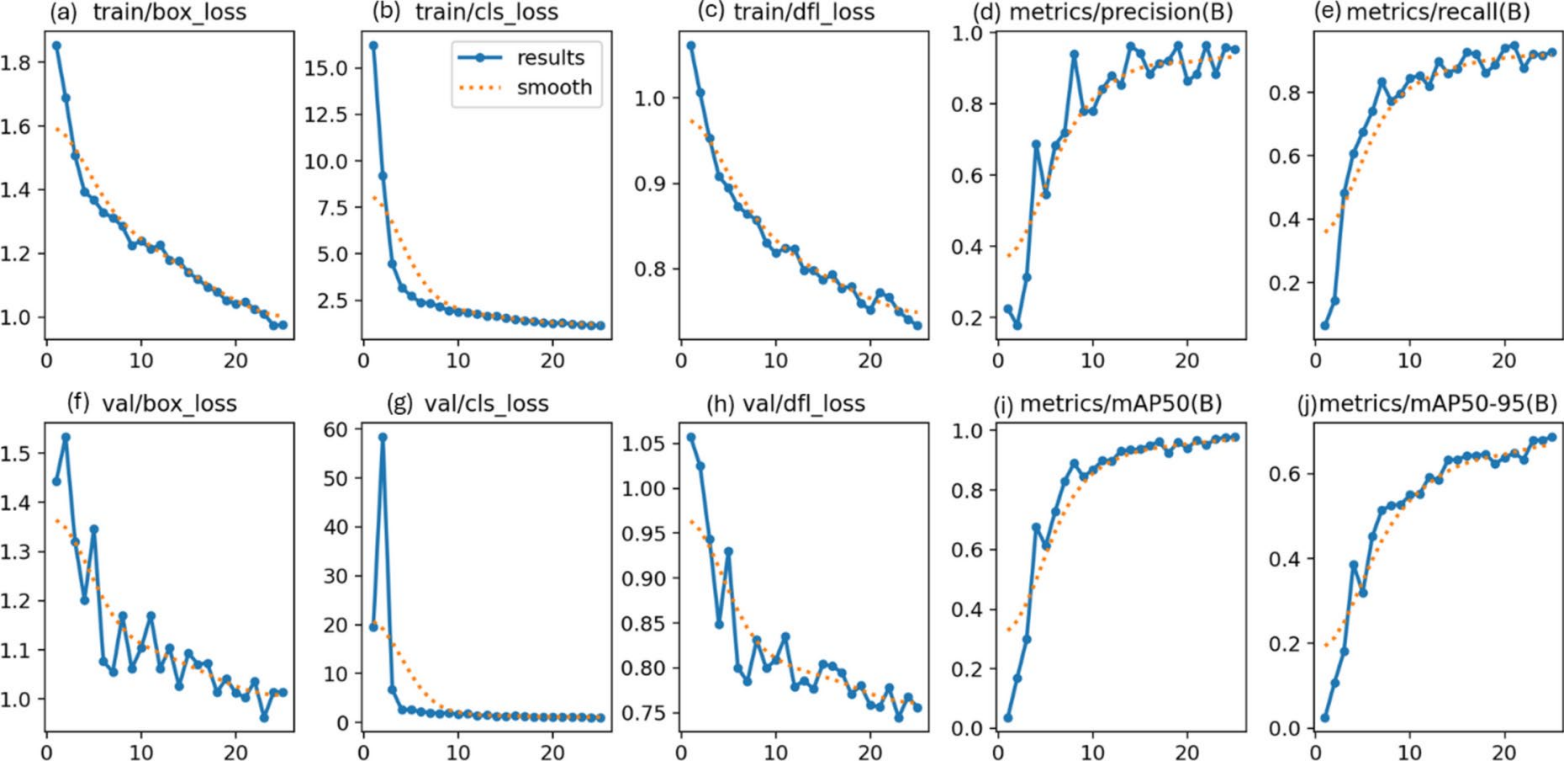
the YOLOv8 optimized by GWO performance metrics on VHR-10 dataset; (**a**) training box loss, (**b**) training cls loss, (**c**) training dfl loss, (**d**) precision curve, (**e**) recall curve, (**f**) validation box loss, (**g**) validation cls loss, (**h**) validation dfl loss, (**i**) training mAP 0.5, (**j**) training mAP 0.5:0.95.

The results in Table 7 show that with no optimization technique applied, the YOLOv7 model obtained a fit score of 0.7. The fit score changed when paired with other optimization techniques; AO and GA approaches achieved a fit value of 0.45, suggesting a great approach. The model’s performance on a range of IoU thresholds may be enhanced by the GWO approach, as evidenced by the method’s top mAP 50:95 scores for training, validation, and testing. It’s crucial to remember that the HPSGWO approach produced a high recall score of 0.85 even if its precision score was comparatively lower at 0.52. This implies that the model is successful in identifying the relevant information in the dataset, even if it can have a larger false positive rate. Among all the optimization techniques applied to YOLOv7, the GWO approach has the lowest fit score (0.39), suggesting the best fit. This shows that, for YOLOv7 on the VHR-10 dataset, the GWO technique could be the best optimization approach. Furthermore, with no optimization technique applied, the YOLOv8 model obtained a fit score of (0.33). The fit score varied when paired with different optimization techniques; among the techniques employed with YOLOv8, the GWO approach achieved the lowest fit score of 0.28. In both training and validation, the GWO technique produced the highest mAP 50:95 scores, indicating that it may be especially useful for enhancing the model’s performance across a variety of IoU thresholds. It’s crucial to remember that the PSO, HPSGWO, ROA, and AO approaches produced excellent precision and recall scores while having a little higher fit score. This suggests that while these models may not have the best-fit score, they are effective at identifying the relevant objects in the dataset and have a lower false positive rate. This shows that even though these models might not match the dataset perfectly, they are good at finding the pertinent items and have a lower false positive rate. In addition to the previously mentioned findings, a visualization showing precision-recall curves and other metrics like losses and mAPs has been done to guarantee the positive effect of the metaheuristic optimization techniques on the precision and effectiveness of the object detection systems used with the VHR-10 dataset. Figure 16 represents the YOLOv7 with GWO optimizer performance metrics (best optimized YOLOv7), and Fig. 17 the YOLOv8 with GWO optimizer performance metrics (best optimized YOLOv8).

**Fig. 18:**
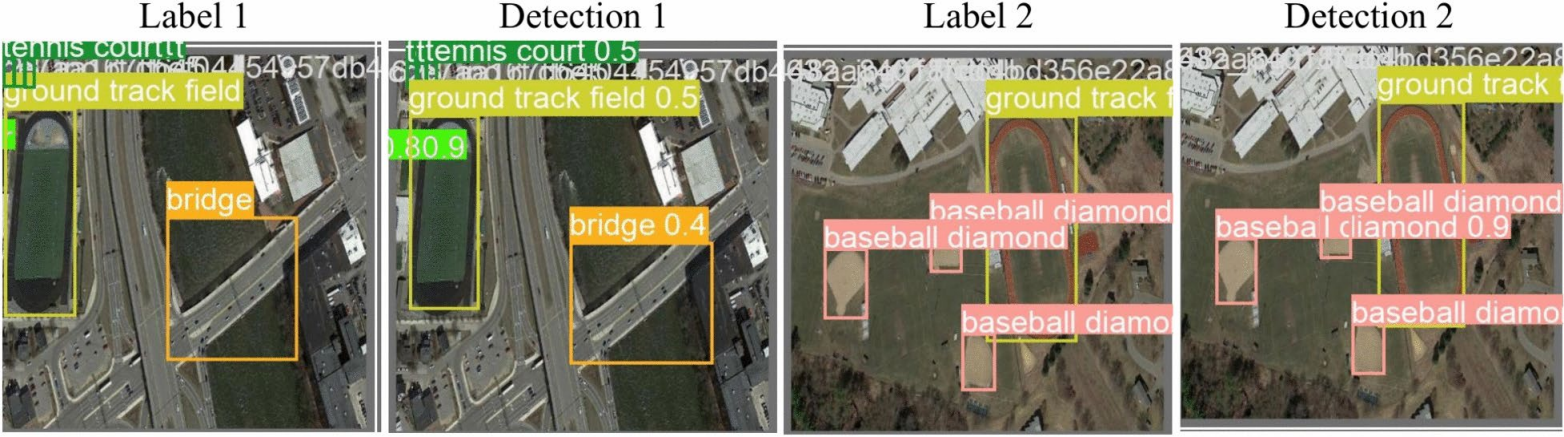
The detection results of the VHR-10 dataset test samples using YOLOv8 optimized by GWO.

In summary, the model’s performance metrics were greatly enhanced by the use of metaheuristic optimization techniques with YOLOv7 and YOLOv8, with the GWO method exhibiting the most potential. Furthermore, the experiment ensures that applying metaheuristic optimization may have a great enhancement in the overall system performance of YOLO algorithms. Figure 18 represents the detection results of the VHR-10 dataset test samples using YOLOv8 optimized by GWO.

In addition to the previous analysis of the experiment results, the outcomes of the two experiments indicate that the Grey Wolf Optimizer (GWO) outperforms other metaheuristic methods in terms of enhancing YOLO algorithms for object detection in remote sensing images which can be referred to the different metaheuristic algorithms methods of navigating and making use of the search space, which is why different metaheuristic algorithms yield diverse results, and The GWO’s hierarchical search strategy, which successfully strikes a balance between exploration and exploitation. Fine-tuning YOLO models is one of the difficult optimization tasks that GWO is most suited for because of its flexibility, ease of use, and dynamic parameter management. Through the utilization of these benefits, GWO can keep clear of local optima and accomplish precise and effective object detection in distant sensing images.

### Study limitations

The major potential restrictions and difficulties that the suggested model could encounter have been outlined in this section:

1. **Huge dataset sizes and computational complexity**: Handling large datasets poses computational difficulties that affect the effectiveness of the model and demand resources for both training and inference.
2. **Errors in the original boundary boxes:** Improving the accuracy and dependability of the model requires fixing errors in the detection annotations.
3. **Optimization challenges**: By concentrating on elements like convergence improvement and overall generalization enhancement, it is imperative to overcome optimization challenges to ensure a practical and effective deployment.

## Conclusion

This study demonstrates the relevance and possibility of applying metaheuristic and hybrid metaheuristic optimization strategies to improve the performance of the YOLOv7 and YOLOv8 algorithms for object detection in remote sensing data. The comprehensive comparison of six alternative metaheuristic optimization algorithms (GWO, PSO, GA, ROA, AO, and HPSGWO) demonstrated their efficacy in enhancing precision, recall, and mean average precision (mAP) of YOLO algorithms at various stages of the machine learning process. The study indicated that the GWO-optimized YOLOv7 model and the HPSGWO-optimized YOLOv8 model performed better on the RSOD dataset, as reflected by higher mAP scores. Similarly, in the VHR-10 dataset, GWO-optimized versions of YOLOv7 and YOLOv8 had the best scores, demonstrating the durability and applicability of the GWO optimization approach for the used datasets.

These findings support the hypothesis that metaheuristic optimization may considerably improve the performance of object identification algorithms when dealing with high-dimensional, non-linear remote sensing data. The findings not only enhance the understanding of the complex interaction between optimization approaches and YOLO algorithms, but they also give practical insights into how to apply these techniques in real-world applications such as urban planning, environmental monitoring, and surveillance.

Future work might include exploring additional optimization techniques, developing new hybrid models, applying models to more datasets, investigating real-time implementation and scalability, integrating with other deep learning techniques, and considering extended evaluation metrics.

## Data Availability

The data that support the findings of this study are available within this article.

## Declarations

### Data attribute

All the datasets in this article are publicly available and need no permissions. NWPU VHR-10 dataset is available at: https://github.com/chaozhong2010/VHR-10_dataset_coco. RSOD dataset is available at: https://github.com/RSIA-LIESMARS-WHU/RSOD-Dataset-.
